# Spatially interactive modeling of land change identifies location-specific adaptations most likely to lower future flood risk

**DOI:** 10.1038/s41598-023-46195-9

**Published:** 2023-11-01

**Authors:** Georgina M. Sanchez, Anna Petrasova, Megan M. Skrip, Elyssa L. Collins, Margaret A. Lawrimore, John B. Vogler, Adam Terando, Jelena Vukomanovic, Helena Mitasova, Ross K. Meentemeyer

**Affiliations:** 1 Center for Geospatial Analytics, North Carolina State University, Raleigh, NC USA; 2 Southeast Climate Adaptation Science Center, U.S. Geological Survey, Raleigh, NC USA; 3 Department of Applied Ecology, North Carolina State University, Raleigh, NC USA; 4 Parks, Recreation and Tourism Management, North Carolina State University, Raleigh, NC USA; 5 Department of Marine, Earth and Atmospheric Sciences, North Carolina State University, Raleigh, NC USA; 6 Department of Forestry and Environmental Resources, North Carolina State University, Raleigh, NC USA

**Keywords:** Environmental sciences, Natural hazards, Climate-change adaptation, Climate-change impacts

## Abstract

Impacts of sea level rise will last for centuries; therefore, flood risk modeling must transition from identifying risky locations to assessing how populations can best cope. We present the first spatially interactive (i.e., what happens at one location affects another) land change model (FUTURES 3.0) that can probabilistically predict urban growth while simulating human migration and other responses to flooding, essentially depicting the geography of impact and response. Accounting for human migration reduced total amounts of projected developed land exposed to flooding by 2050 by 5%–24%, depending on flood hazard zone (50%–0.2% annual probability). We simulated various “what-if” scenarios and found managed retreat to be the only intervention with predicted exposure below baseline conditions. In the business-as-usual scenario, existing and future development must be either protected or abandoned to cope with future flooding. Our open framework can be applied to different regions and advances local to regional-scale efforts to evaluate potential risks and tradeoffs.

## Introduction

Projecting human mobility and shifts in development patterns in response to future flooding is crucial for anticipating the need for policies and/or investments that protect lives, livelihoods, and property^1^. Geospatial modeling offers opportunities to assess conditions under future scenarios of climate and land change. Here we present the first open, spatially interactive (i.e., what happens at one location affects another) modeling framework that simulates plausible human responses to flood risk and integrates all three components of flood risk^2^ relevant to policy-making: exposure, hazard, and vulnerability. Flood risk, or the probability of flood damage at any particular time and place, depends on all three. For the purposes of policy-making, patterns in urban development influence exposure, or the amount of infrastructure that could be exposed to damage if a flood occurred. Hazard relates to the likelihood and intensity of floodwaters, and as sea levels rise and riverine flooding increases due to global climate change, the spatial area of flood hazard also increases. Lastly, vulnerability relates to the capacity of a population to reduce their exposure or diminish hazards through adaptive response; seawalls, for example, are intended to diminish hazard by preventing floodwaters from reaching protected areas, and elevating homes or retreating from hazardous areas reduces exposure, but the resources of populations dictate their capacity to enact these responses. Previous studies have primarily focused on modeling only one or two of the three components of flood risk, and few have integrated all three, with available modeling methods constrained in terms of accessibility, scalability, and generalizability^2–4^.

For example, to predict future flood exposure, some studies have modeled changes in land use^5^ or urban growth^6^ and predicted future population size^7^ but have not considered changes in flood hazard caused by climate change scenarios. Geophysical models that predict changes in hazard due to sea level rise and flooding, meanwhile, have not accounted for future changes in urban growth and therefore increases in human exposure^8,9^. Both components––changes in exposure and changes in flood hazard––should be modeled in concert to better predict risk, but such modeling efforts must also account for vulnerability and human adaptive responses to flood hazard.

Failing to incorporate human adaptation responses into flood risk assessments can grossly overestimate predictions of flood damage risk^10–12^. Yet, projections of the number of people exposed to future flooding typically have not considered adaptation to flood risk^13–16^ or site-specific adaptation strategies based on socio-economics, or alternative “what-if” scenarios^17^. When modeling has considered adaptation, spatially interactive^18^ patterns and processes of future urban expansion have not been considered; instead, population and urban growth have been represented by increasing population density within existing urban areas, without considering that what happens at one location affects another, limiting prediction of the location and amount of flood-exposed area^19–24^. Additionally, methods that focus on individual-level adaptive behavior tend to be computationally demanding, applicable only to specific locations, and challenging to scale up for broader contexts (e.g., agent-based models^2,20,21^ or dynamic systems models^25^). Important recent contributions have investigated the combined impact of urban expansion and global adaptation policies (e.g., coastal setback scenarios^26^) in response to future flood hazard. However, it is crucial to acknowledge that communities vary in their vulnerability to flood hazard and their capacity to adapt^27^. Therefore, modeling approaches need to incorporate these factors to ensure accurate assessments and the generation of relevant scenarios. The composition of at-risk (and near-risk) communities is also changing due to climate gentrification^28^, whereby properties become more valuable when they can accommodate development under climate change and lower-income residents are priced out.

To address the need for an accessible, scalable, and generalizable predictive model that integrates all three components of flood risk––exposure (patterns in urban development within floodplains), hazard (increases in flooding due to climate change), and vulnerability (capacity for adaptive response)––we built on an existing open-source land change model called FUTURES (FUTure Urban-Regional Environment Simulation^29^). The new version (FUTURES 3.0) integrates dynamic flood event modeling and human adaptive response to project spatially interactive patterns of urban growth via population redistribution and adaptation in the face of future urbanization and climate change. In the context of this study, human adaptive response is defined as the continuum of local efforts to reduce exposure to flood hazard, broadly categorized as either migration or in-situ measures; migration represents retreat and resettlement with subsequent conversion of formerly occupied land into abandoned land, and in-situ measures include elevation of structures, nature-based solutions, and coastal hardening.

**Figure 1:**
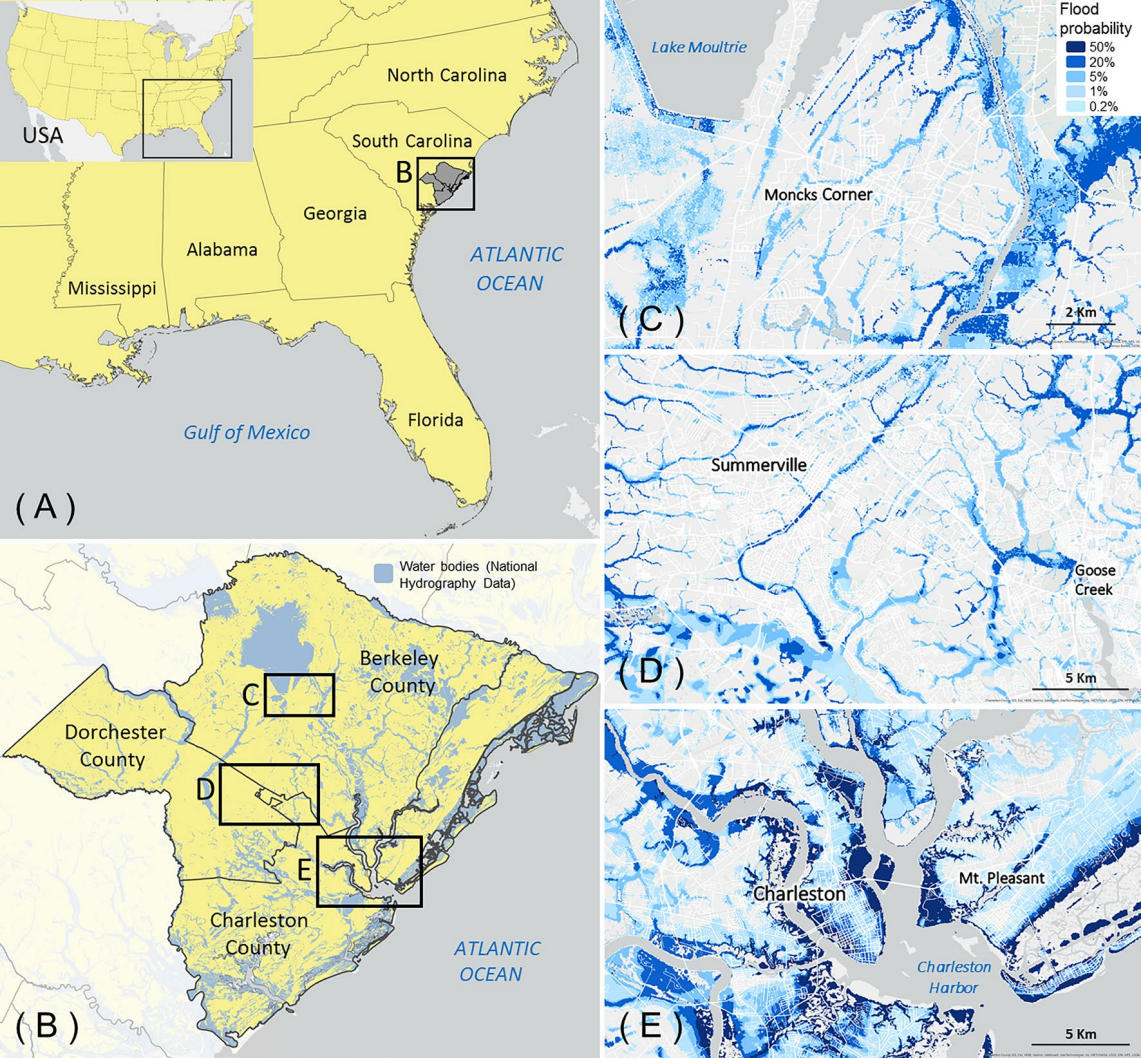
Test case location in the Southeast U.S. (**A**) comprising three coastal plain counties in the Lowcountry of South Carolina (**B**) with detailed maps of annual flood probability in the Towns of Moncks Corner (**C**) and Summerville (**D**) and the City of Charleston (**E**). Detail maps (**C**, **D**, **E**) display anticipated flood hazard due to fluvial, pluvial, and coastal flooding under a moderate greenhouse gas emissions scenario (Representative Concentration Pathway [RCP] 4.5) by 2050.

To demonstrate this new modeling framework, we selected a test case location in the Southeast U.S. comprising three counties near the fast-growing Charleston Metropolitan Area of the coastal South Carolina Lowcountry (Fig. 1). The region is arguably at the leading edge of climate adaptation response in the U.S., given its vulnerability to flooding, historical efforts to reduce flood impacts, and future plans to continue responding to flood hazards^30,31^. This low-lying region borders the Atlantic Ocean, contains dozens of rivers, and is experiencing rapid urbanization, placing its natural amenities and cultural ecosystem services under “coastal squeeze” from sea level rise and urban development^32^. The Lowcountry is increasingly prone to flooding from storms, high tides (a.k.a., sunny day flooding), and sea level rise, and its human population will become progressively exposed with more urbanization. Concurrently, regional land subsidence^33^ and dredging efforts to expand navigable channels^34^ exacerbate flood risks. By 2050, the City of Charleston is expected to experience up to 233 high-tide flood events per year (i.e., following a global mean sea level rise scenario of 0.37 m by 2050), compared to only 50 in 2016 and 14 in 2019^35^. The Charleston Metro Area is also growing three times faster than the U.S. average^36^, putting pressure on the city’s infrastructure and capacity to effectively manage coastal flooding. Lower-density suburban development from regional growth encircles the Charleston Metro Area, but a considerable amount of the Lowcountry remains rural. This urban-to-rural continuum permits investigating flooding and sea level rise impacts, and potential responses, across a socio-economically diverse region in which we have previously worked^32^. The area also well represents the vulnerabilities and capacities of communities experiencing the greatest impacts of sea level rise and frequent flooding^34,37^.

## Modeling approaches

We compared the amount of developed land area predicted to be exposed to future flooding using three modeling approaches, to demonstrate the necessity of including all three components of flood risk––exposure, hazard, and vulnerability––in one modeling framework. We call the three modeling approaches static development, dynamic development, and climate-aware development (Supplementary Information [SI] Appendix, Fig. S1). All approaches used temporally and spatially explicit flood hazard information about fluvial (from rivers), pluvial (from rainfall), and coastal (from sea level rise and storm surge) flooding in 2020, 2035, and 2050^38^; we accounted for increasing flood probabilities over time, following a stabilizing greenhouse gas emissions scenario (Representative Concentration Pathway [RCP] 4.5 climate change scenario). The dynamic development and climate-aware development modeling approaches are probabilistic, and we ran each type for 50 stochastic iterations from 2020 through 2050 at annual time steps. Further details about the models and data are provided in “Methods”.

The *static development* approach assumes that the built landscape, as of 2019, remains constant; there is no urbanization or population growth into the future. It models the current infrastructure and developed areas exposed to future flooding but does not account for future growth or humans’ adaptive response to this increasing hazard.

The *dynamic development* approach accounts for future population growth and urban development, modeling the concurrent growing footprints of flooding and infrastructure to predict future flood exposure. Future population projections are based on a moderate socio-economic scenario (Shared Socioeconomic Pathway 2; SI Appendix, Table S2), and development patterns assume the continuation of historical growth trends. Humans’ adaptive response to flooding is not incorporated.

The *climate-aware development* approach integrates the combined effects of spatially interactive urbanization processes and human adaptation to flooding to predict exposure. It uses a flood response function that relates adaptive capacity and level of flood damage sustained (see Methods). This function delineates three response state spaces––stay trapped, retreat, or protect and armor––based on published literature^11,27,39–45^. For our test case, we devised a plausible business-as-usual “reactive” flood response function, wherein residents are assumed to adapt to threats as they occur, without incentives or policies in place to affect outcomes. This “reactive” response scenario assumes that the residents with the lowest vulnerability are most likely to protect and armor, whereas those with the highest vulnerability are more likely to either retreat or stay trapped. We defined a flood damage threshold above which retreat is likely inevitable regardless of adaptive capacity (75% of the structure’s value; see Methods section Adaptive Response). Other shapes of this function are possible, parameterized to explore scenarios of response and represent the inclinations of different communities or the influence of policies. We present four additional response functions (managed retreat, resist, polarized population, trapped population) to assess the sensitivity of the geography of simulated outcomes to model parameters (e.g., threshold of damage above which residents retreat). This not only demonstrates the flexibility of our modeling approach for evaluating policy interventions but also highlights its capacity to facilitate formulating locally appropriate solutions across a range of response scenarios (see “What-if” Policy Interventions and Flood Response Scenarios).

## Results

**Figure 2:**
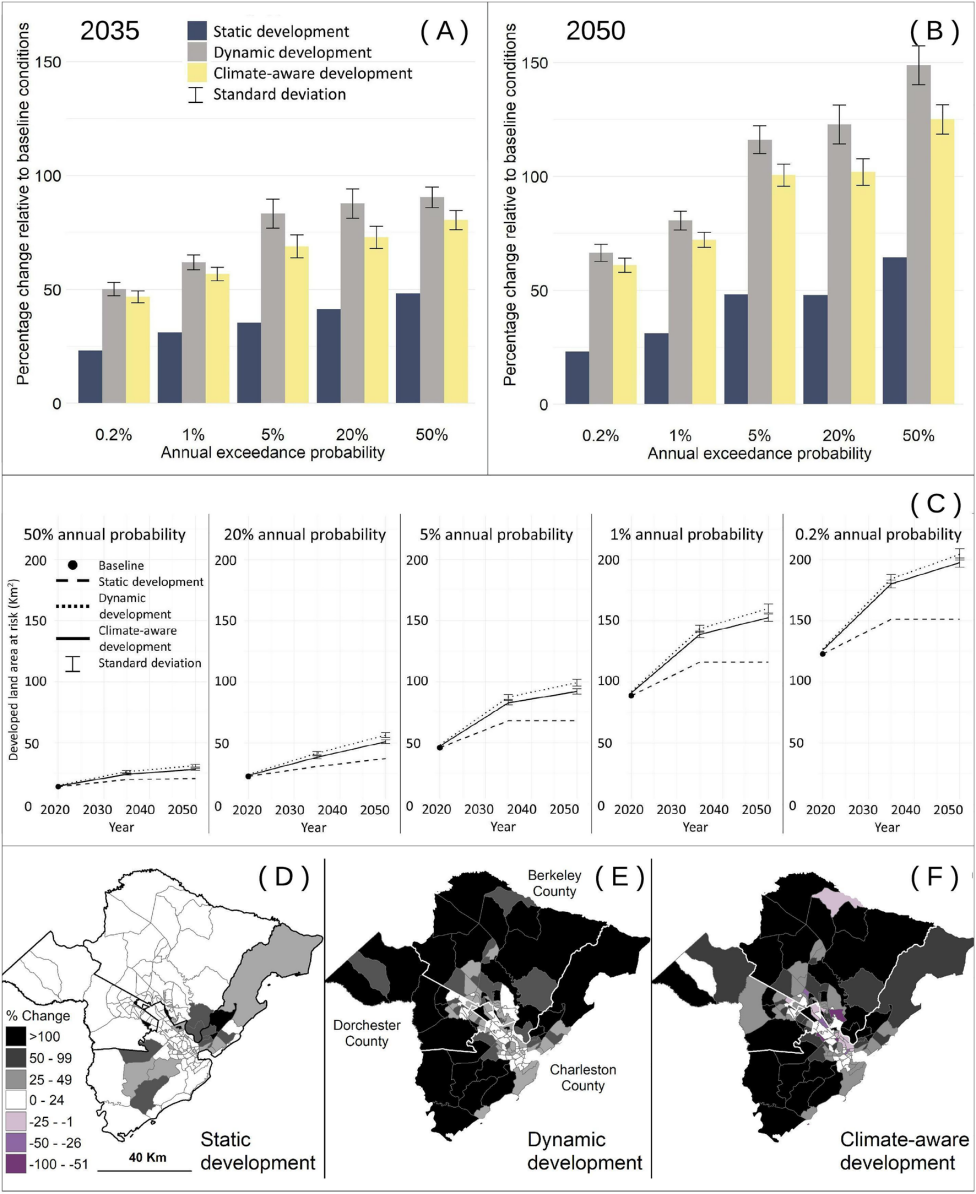
Percentage change and total developed land exposed to future flooding relative to baseline conditions (i.e., 2019 development, 2020 flood hazard). Bar graphs display anticipated percent increases in developed land exposed to a 0.2% (500-year floodplain), 1% (100-year floodplain), 5% (20-year floodplain), 20% (5-year floodplain), and 50% (2-year floodplain) annual chance of flooding by 2035 (**A**) and 2050 (**B**) for the three modeling approaches (static development, dynamic development, and climate-aware development [“reactive” response scenario]). The difference in exposed developed land area between dynamic development and climate-aware development is attributable to retreat. Line graphs (**C**) display the estimated cumulative developed land area (km^2^) within different hazard zones (50%, 20%, 5%, 1%, and 0.2% annual chance of flooding) through time and by modeling approach. The standard deviations displayed for dynamic development and climate-aware development were derived from the 50 stochastic urban growth simulations; static development has no standard deviation, because it represents only 2019 development. Maps (**D**–**F**) show percentage change in developed land exposed to future flooding (i.e., annual flood probability of 0.2% by 2050) by census tract unit across the three-county test case location in South Carolina, U.S. Percentage change for the static development (**D**), dynamic development (**E**), and climate-aware development (F; “reactive” response scenario) modeling approaches.

Our climate-aware modeling approach using the “reactive” response function predicted an intermediate amount of total developed land exposed to future flooding in 2035 and 2050 (Fig. 2) compared to the static and dynamic development modeling approaches. By incorporating the departure of some residents (retreat and resettlement with subsequent conversion of developed land into abandoned land), the climate-aware approach produced lower estimates of exposed development than the dynamic development approach. The static development approach predicted the least total development land exposed to future flooding, but nonetheless predicted an increase in exposure compared to baseline because of increases in flood hazard due to climate change. The dynamic approach predicted the most total exposure (~ 82 km^2^ or 66% more exposed developed land than baseline conditions by 2050), due to both increases in urban growth and flood hazard, but no incorporation of adaptive response.

The climate-aware modeling approach revealed that locations with a 5% or greater chance of flooding in any given year are highly likely to experience retreat (lower percentage change in exposed developed land relative to baseline conditions than the dynamic development approach; Fig. 2A,B). By 2050, the difference between the dynamic and climate-aware modeling approaches was greatest for areas with a 20% annual chance of flooding (inside the 5-year floodplain). This difference in exposed developed land area between the two approaches is attributable to retreat.

In terms of total developed land area exposed to flooding (Fig. 2C), both the climate-aware and dynamic modeling approaches predicted similar amounts of development through time and for all flood probabilities. However, the stochastic challenges (see Methods) and reactive response function used in the climate-aware approach simulated the implementation of adaptation strategies to withstand flood damage in those floodplains. The consistently lower amount of at-risk developed land predicted by the climate-aware approach relative to the dynamic approach represents the retreat response.

Retreat predicted by the climate-aware approach varied spatially, with some census tracts resulting in a lower amount of developed land exposed to flooding than current (baseline) values (Fig. 2D–F). In the absence of any measure to control flooding, the census tracts with negative values (Fig. 2F) represent areas where residents are likely to retreat. In the static (Fig. 2D) and dynamic (Fig. 2E) approaches, the amount of developed land exposed to flooding only increased.

**Figure 3:**
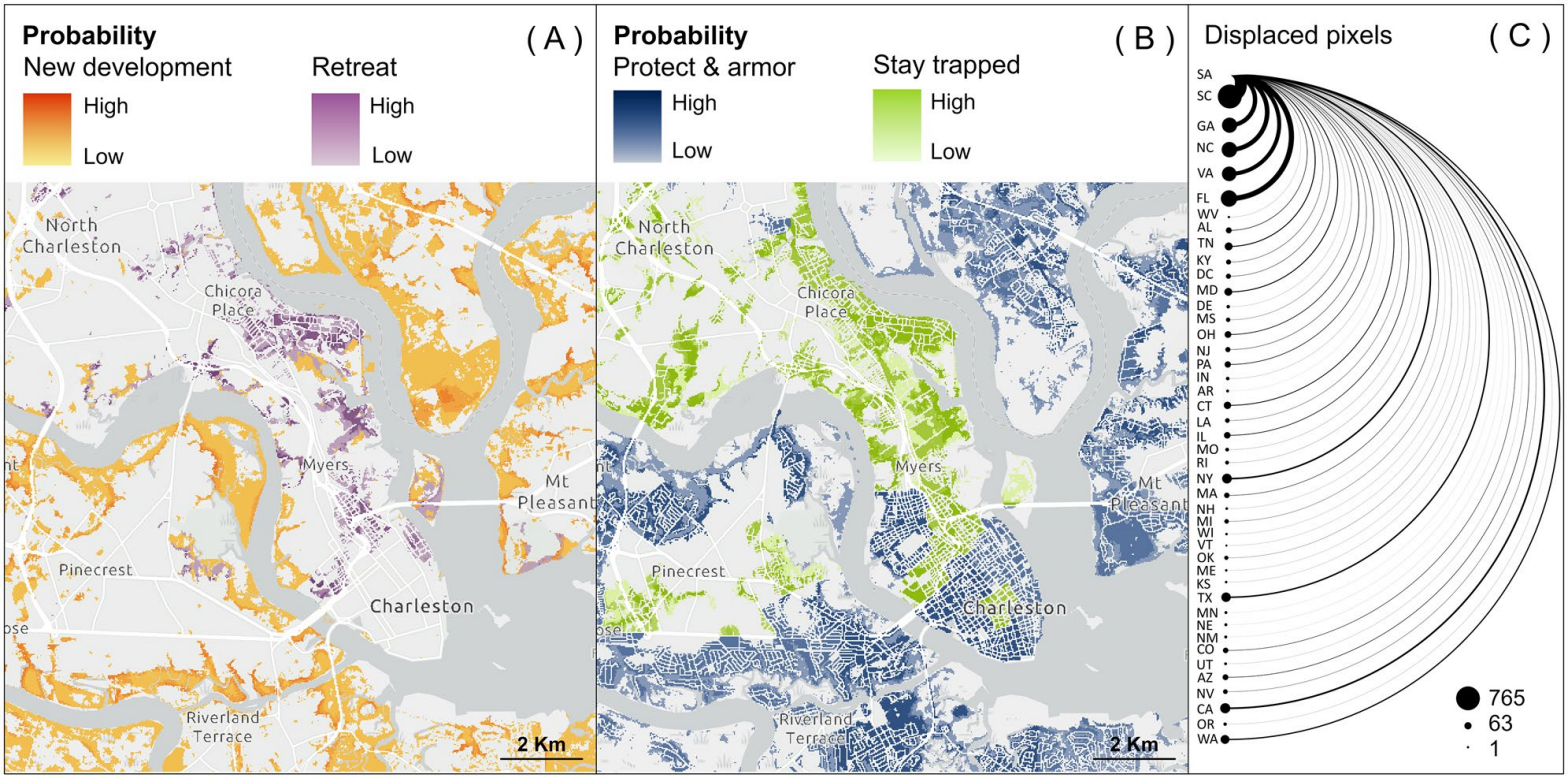
Geography of impact and response. Locations and probabilities by 2050 of simulated new development or retreat (**A**) and protect and armor or “stay trapped” (**B**) across the Charleston Metropolitan Area, South Carolina (U.S.); protect and armor is in response to a 1% annual chance of flooding (100-year floodplain). Probability maps (**A**–**B**) are derived from 50 stochastic urban growth simulations computed with the climate-aware modeling approach for a “reactive” response scenario. Likely destinations of residents from the three-county study area (SA) that resettled outside of the study area due to “retreat” (**C**); numbers of displaced pixels are averaged across the 50 stochastic simulations. The thickness of lines and size of dots indicates the relative number of developed pixels displaced from the study area to a new destination within South Carolina (SC) or another state (two-letter abbreviations). State abbreviations are ordered top to bottom by increasing distance from the study area.

The spatial resolution of our simulations (30-m) permitted identifying locations where new development is expected (Fig. 3A) and where retreat (Fig. 3A) or protect and armor adaptation or staying trapped (Fig. 3B) are likely, given the “reactive” response function used in our model (see Methods for details). Our results indicate that a considerable amount of land in the three-county study area is projected to be newly developed (approximately 121 km^2^, SD = 29 km^2^) in flood-prone locations by 2050. Consequently, measures aimed at safeguarding this additional development from potential flood damage may be required in the future. A large portion of existing and future development in the study area would need to be protected (125 km^2^, SD = 43 km^2^) by 2050 or abandoned (through voluntary or forced retreat; 11 km^2^, SD = 13 km^2^) to adapt to future flood hazard, while localities unable to protect and armor or retreat will remain vulnerable to subsequent flood events (stay trapped; 31 km^2^, SD = 3 km^2^; SI Appendix, Fig. S2).

Our model predicted that most (approx. 76%) of the residents expected to retreat from the three-county study area will likely relocate within it (SI Appendix, Table S1). Of the approximately 24% that will leave the area, most will likely relocate elsewhere within South Carolina (27%) or to nearby southern states (Fig. 3C), particularly Florida (12%), Georgia (10%), North Carolina (10%), and Virginia (9%). However, a total of 42 states other than South Carolina are likely to receive residents who retreat, including states as far away as Washington and Oregon.

### “What-if” policy interventions and flood response scenarios

**Figure 4:**
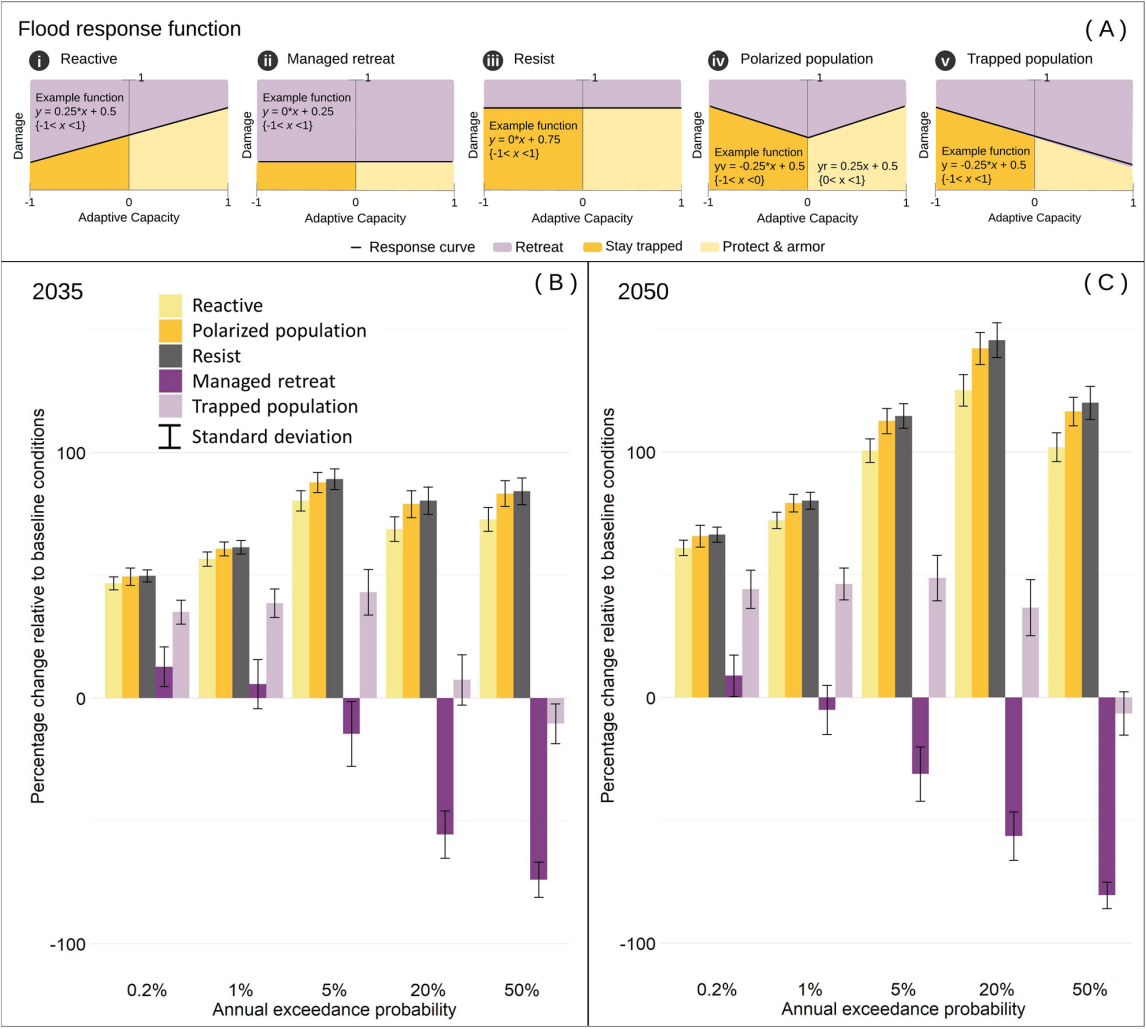
“What-if” scenarios of policy interventions. Additional to the “reactive” response scenario (**A.i**; displayed here for reference), we computed four alternative scenarios: “managed retreat” (**A.ii**), “resist” (**A.iii**), “polarized population” (**A.iv**), and “trapped population” (**A.v**), with the climate-aware development modeling approach. Bar graphs display anticipated percent changes in developed land exposed to a 0.2% (500-year floodplain), 1% (100-year floodplain), 5% (20-year floodplain), 20% (5-year floodplain), and 50% (2-year floodplain) annual chance of flooding by 2035 (**B**) and 2050 (**C**) as relative to baseline conditions (i.e., 2019 development, 2020 flood hazard). Standard deviations were derived from 50 stochastic urban growth simulations.

Besides a “reactive” response function, we parameterized four others, which we called managed retreat, resist, polarized population, and trapped population. We utilized these four additional parameterizations (Fig. 4) to assess the impact of varying shapes of the adaptive response function on the geography of simulated outcomes. This involved testing different thresholds of damage at which residents retreat. “Managed retreat” results in the lowest amounts of developed land exposed to future flooding compared to baseline conditions and all other modeled scenarios, particularly in areas with annual flood probabilities greater than 1% (100-year floodplain; Fig. 4B,C). Managed retreat is the only scenario that predicts a decrease in the amount of total developed land in flood-exposed areas over time below baseline conditions (SI Appendix, Fig. S3). Furthermore, managed retreat resulted in a greater number of census tracts overall demonstrating some level of retreat and pointing to a regional pattern (SI Appendix, Fig. S4). “Resist” revealed the highest percent increases in developed land exposed to future flooding (Fig. 4B,C). While estimated exposure for the resist scenario is highly comparable to results associated with the dynamic development modeling approach (SI Appendix, Fig. S3), a few census tracts with negative values (SI Appendix, Fig. S4) show areas where residents experiencing high levels of flood damage are likely to retreat. With a “polarized population,” the probability of retreat is greater than in the resist scenario, but overall smaller than in the reactive scenario (Fig. 4A). Estimated exposure for a polarized population falls between those two scenarios (Fig. 4B,C and SI Appendix, Fig. S3), while the spatial pattern of estimated exposure by census tract closely resembles the patterns presented by the resist scenario (SI Appendix, Fig. S4). Compared to all scenarios, except “managed retreat”, “trapped population” results in considerably lower levels of exposure across all hazard zones (Fig. 4B,C and SI Appendix, Figs. S2 and S3). The response function for this scenario decreases retreat for more vulnerable communities and increases it for less vulnerable ones; in our study area, flood-prone areas such as high-value waterfront property predominantly exhibit lower vulnerability (SI Appendix, Fig. S7). Consequently the “trapped population” scenario concentrates the high probability of retreat in less vulnerable census tracts, including waterfront areas (SI Appendix, Fig. S4).

## Discussion

Our open science approach to developing FUTURES 3.0 responds to the global need for flexible and transferable modeling frameworks that can integrate three critical components of flood risk relevant to policymaking^1^. We simultaneously modeled urban growth, increases in flood hazard due to climate change, and human adaptive response to flooding to estimate the future flood exposure of built infrastructure. Given that urban areas continue to expand, that flood hazards are intensifying under climate change^9,38^, and that people (if capable) are likely to try to reduce their flood risk or prevent further damage to their properties^46^, integrating all three components is important when estimating future risk and exposure.

Anticipating human responses to flood hazards or damage is a challenging task, given that the past may not be a good proxy for the future and that few studies have retrospectively examined human decision-making in the face of flood risk or damage^47^. Prior research seeking to predict responses have focused on simulating adaptation decisions based on cost–benefit analyses^19^ or bounded rationality of individual agents^2,20–22^. Our scenario-based approach using plausible response functions is presented as an alternative, to overcome the difficulties in accounting for “irrational” decision-making or the non-economic factors that influence a choice in traditional cost–benefit approaches^20^ and the computational expense and limited transferability of agent-based modeling. In this way, scenario modeling permits conducting simulation experiments to test approaches for efficiently and equitably allocating resources for adaptation. These experiments can be used to iteratively test assumptions or predict outcomes based on lessons learned from regional-scale analysis of flood management policies (e.g.^48,49^), local case studies of damage and adaptive capacity (e.g.^43,50^), or community-specific plans (e.g.^51,52^). FUTURES 3.0 enables the use of customizable flood response functions to capture the range of human behaviors likely for specific areas, providing an array of alternative scenarios of what may happen under different assumptions of political or economic conditions. Response function parameterization will determine the simulated amount of development exposed to flooding in response to policy interventions (see “What-if” Policy Interventions and Flood Response Scenarios).

Of particular note in the flood response functions is the option to retreat, which has a complicated relationship with social vulnerability and equitable adaptation^44,46^. In our test case, we assumed retreat to be voluntary for the least vulnerable census tracts and forced for the most vulnerable ones, reflecting current inequities, but the FUTURES 3.0 model does not distinguish between these two types of retreat. Generally, more affluent and privileged residents and homeowners have a greater range of adaptation options. They are more likely to possess, or be able to acquire, the resources needed to adapt properties (e.g., elevate structures), and they are in a better position to navigate securing a buyout and are more likely to be successful in a new location^41,42,46,53–55^. Importantly, buyout programs currently benefit only individual homeowners and do not facilitate the relocation of whole communities; existing policies therefore disregard the social ties and connections to place integral to both individual and group identity and success for many members of tight-knit and Indigenous communities^44^. Without the resources to protect themselves and maintain social support systems and livelihoods, underprivileged residents and renters may be forced to abandon their homes only after severe flooding results in an untenable living situation, exacerbating their social vulnerability.

By integrating retreat and resettlement into our model, we were able to project where displaced residents are likely to go (Fig. 3C). Our results are comparable to those of other researchers^18^ that used similar methods to predict both intrastate and interstate migration. Our modeling predicts new “destination” communities to concentrate across the Southeast (i.e., South Carolina, Florida, North Carolina, and Georgia), but displaced residents are likely to relocate as far west as Washington, Oregon, and California. Destinations carry their own flood risks or other stressors or hazards, however, and it is likely that future adaptation will be necessary to protect both new and established residents. It is also uncertain whether “destination” communities are prepared to receive in-flows of climate migrants^19,21^ and how human movements might impact natural resources (e.g., conservation corridors).

## Study limitations and future work

There are several limitations of our study to consider for future work with FUTURES 3.0. First, for simplicity, we did not simulate restricting development in areas with high flood hazard, i.e., to proactively prevent construction that might require protection or be abandoned in the future. Restricting future development in FUTURES 3.0, however, is possible (e.g., using a spatial protection layer [see^56^ for an example using FUTURES 2.0], or with a flood response function that uses managed retreat) and may be useful for planning^26^ or scenario development that focuses on conservation of wetlands and other open spaces that provide flood protection services. Second, we used historical migration trends (1990–2015) to anticipate where displaced residents migrate, but it is also possible to incorporate “climate-aware” migration trends that anticipate how migration is influenced by climate^57^ and climate change^1,18,20^. Such migration patterns would consider “pulls” to new areas (e.g., economic advantages, natural amenities, and comfortable temperatures) and “pushes” that both cause current residents to leave and dissuade new settlement (e.g., hazards including floods, wildfires, extreme temperatures, and droughts).

Further enhancements to FUTURES will enable more fully integrating how economic considerations impact human development and adaptive behavior. Using the locations of planned new roads or job-creating industries would likely improve predictions of desirable areas for future development. Considering how flood damage influences local or regional economies would capture the “indirect” effects of flooding on a household’s decision to relocate^20,21,58^. Distinguishing between different types of land use in FUTURES’ exposure assessment would more clearly indicate where homes versus businesses are experiencing flood damages that could lead to job loss or other forms of economic disruption. Including calculations of adaptation cost may be useful for planning allocation of resources for targeted protection from flood impacts^19,22,23^ as well as for assessing the costs vs. benefits of retreat and/or protective investments.

Integrating the feedback loop between development, protective measures, the natural cycling of water, and flood hazard (e.g., through expanded impervious surface) provides another opportunity for future work. Without interventions or the expansion of infrastructure that can handle larger volumes of stormwater runoff, new development only exacerbates the flood risk of existing development. Capturing this feedback loop in a simulation could be accomplished by integrating hydrologic modeling with FUTURES; the impact on runoff and streamflow of simulated annual development, and benefits of large-scale protective measures (e.g., gray/green infrastructure), would be iteratively estimated, informing changes in flood hazard that would in turn influence adaptation decisions and further development.

## Call to action

Scenario-based models that effectively project future risks are critical to support forward-thinking planning efforts that can anticipate, or avoid, costly interventions and impacts. Flexible models like FUTURES 3.0 can be used to run simulations for any geographic area, using any land use and flood hazard data and a flood response function that can be customized to reflect local or regional conditions. Given that predicting human behavior is inherently difficult and that future climate change will present unprecedented challenges, reducing uncertainty of how, when, and where adaptation strategies could protect lives and livelihoods and prevent future flood impacts will be essential. We are encouraged by recent institutional efforts to support community engagement projects that can help parameterize flood response functions while acknowledging the value of participants’ time, input, and local knowledge through appropriate compensation. Community engagement projects that focus on scenario development and adaptive planning are crucial to envisioning and creating interventions that are more likely to produce just and equitable outcomes. To have the greatest utility for decision-making, these efforts would require integration of all three components of flood risk^2^: exposure (patterns in urban development within floodplains), hazard (increases in flooding due to climate change), and vulnerability (capacity for adaptive response).

## Methods

**Figure 5:**
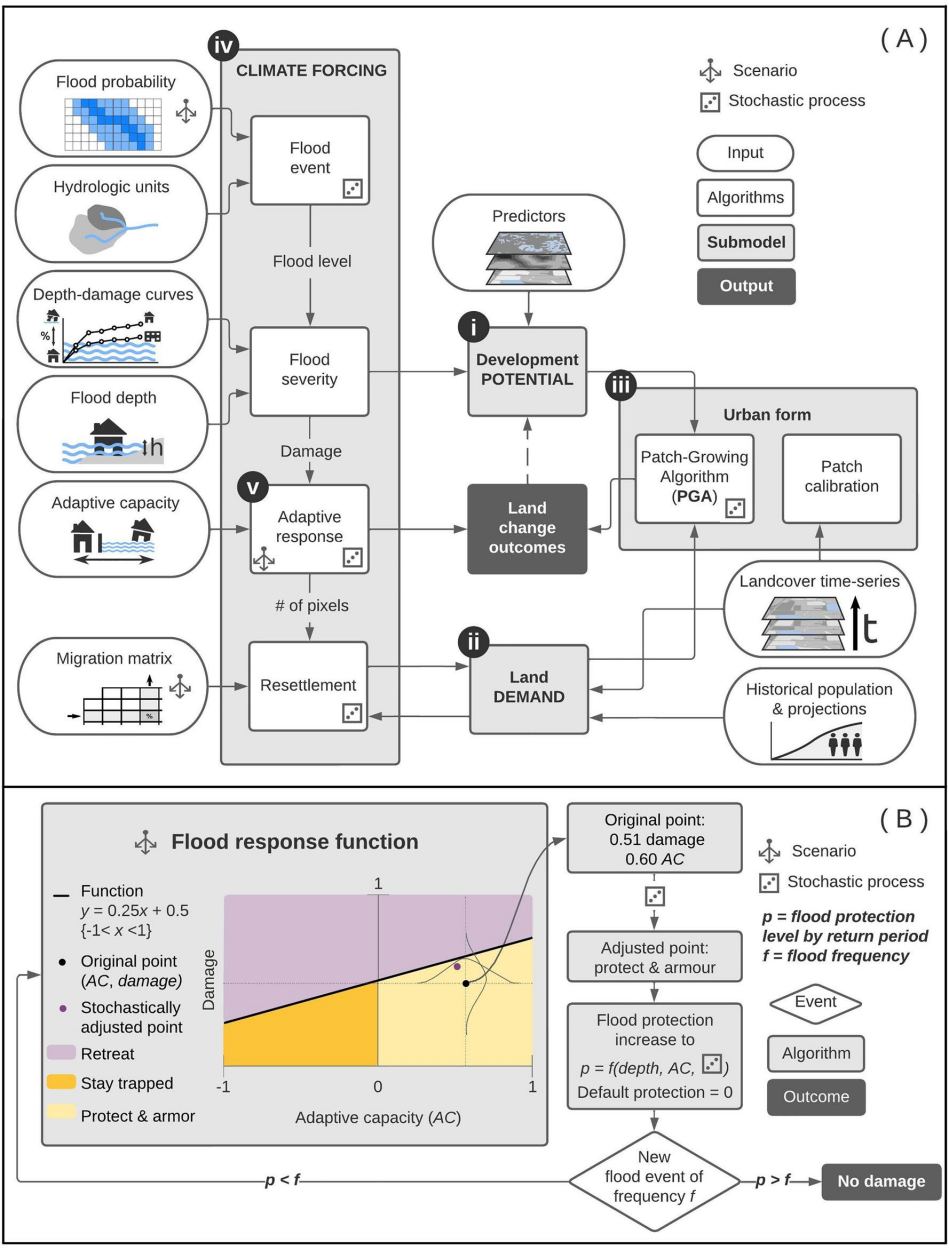
The new FUTure Urban-Regional Environment Simulation framework (FUTURES 3.0). Designed to probabilistically predict new urban development and adaptation measures in response to flood hazard from climate change (**A**). FUTURES simulates spatially explicit patterns of development through the integration of four submodels that consider local site suitability for land change (POTENTIAL; **A.i**), per capita land consumption of a region (DEMAND; **A.ii**), the spatial patterns of urbanization (Patch-Growing Algorithm [PGA]; **A.iii**), and local capacity for adaptive response to flooding due to climate change (CLIMATE FORCING; **A.iv**). Adaptive response (**A.v** and **B**) is based on local estimates of flood probability, level of damage, and adaptive capacity. The core of the CLIMATE FORCING submodel is a flood response function (**B**) that determines whether the residents of a pixel leave the area (retreat) or remain (either stay trapped or protect and armor). The flexibility of FUTURES 3.0 accommodates creating different scenarios and altering the shape of the flood response function to represent community preferences or policy influences. A plausible flood response function (**B**) assumes residents will adapt to threats on an as-needed basis (i.e., “reactive” response scenario).

We developed a new version of the FUTURES land change model (FUTURES 3.0) that can now probabilistically project new urban development while also simulating human migration and other adaptation measures in response to flood hazard from climate change (Fig. 5).

FUTURES is an open source urban growth model designed to address the regional-scale ecological and environmental impacts of urbanization (e.g., assessments of tradeoffs between conservation strategies^32,56^ and ecosystem services^59–61^). It is one of the few land change models that explicitly captures the spatial interactions and processes of development in response to user-specified scenarios^29^. Spatially interactive^18^ urbanization processes consider how the state of one location at a given time influences the state of another location at a subsequent time (e.g., new development in one location affects the likelihood of future development in nearby locations). FUTURES simulates locational interactions and patterns of development by integrating three submodels that consider local site suitability for land change (POTENTIAL^62^; Fig. 5A(i), per capita land consumption of a region (DEMAND^63^; Fig. 5A(ii), and the spatial patterns of urbanization (PGA^64^, or Patch-Growing Algorithm; Fig. 5A(iii)^29,65^. The PGA stochastically assigns placement of discrete spatial objects (patches) of developed land across the POTENTIAL surface, based on the study area's historical trends in the size and shape of land change events. At each time step, the POTENTIAL surface determines whether the patch results in land conversion, and the quantity of new patches is directed by DEMAND. The PGA allows integration of policy-oriented, user-defined parameters to explore alternative development scenarios. FUTURES is publicly available through GRASS GIS and GitHub^65^, has been validated as accurately capturing the spatial configuration of new development^60,66,67^ (see subsection FUTURES 3.0 Validation), and has been used or cited in over 100 land change studies.

FUTURES 3.0 is a new version of the model introducing the CLIMATE FORCING submodel [Fig. 5A(iv)] to estimate the probability that a developed location (e.g., a 30-m pixel) will experience flood damage and its likely adaptation response (protect and armor, retreat, or stay trapped). Adaptive response [Fig. 5A(v)] is based on flood probability^38^, level of damage (see subsection Adaptive Response for details about building structure depth-damage functions), and local estimates of adaptive capacity (i.e., a continuum of low to high vulnerability based on socioeconomic data from the Centers for Disease Control and Prevention, CDC^68,69^).

The CLIMATE FORCING submodel integrates current and future flood probability and flood depth data (see “Data Sources” below) with the adaptive capacity of developed pixels (see “Adaptive Response” below) to probabilistically predict flood severity and the response evoked by simulated damage in a developed pixel. The submodel also predicts the within- or between-county destinations of displaced residents (following methods by^17^). FUTURES is a stochastic model and can generate hundreds of simulations of flood responses at yearly time steps. We programmed the PGA to run 50 independent stochastic iterations of FUTURES 3.0 from 2020 through the year 2050, employing a consistent setup. Through these iterations, we probabilistically predict where new development will occur and where adaptation interventions or the inability to act (staying trapped) is more likely to occur.

### FUTURES parameterization

We parameterized FUTURES following methods detailed by^29,65^. In FUTURES, the POTENTIAL submodel estimates local site suitability for urbanization as a function of hypothesized environmental, infrastructural, and socio-economic predictors. We included in the POTENTIAL submodel the predictor variables that significantly explained land change (i.e., from non-urban to urban) during a reference period (2001–2019) while maintaining parsimony and predictive accuracy (SI Appendix, Table S2). We assumed that urbanization processes can vary from region to region, and therefore, FUTURES uses a multilevel model structure (at the county level in this research) that accounts for the spatial nonstationarity of drivers of urban development (SI Appendix, Table S3). We assumed no new development occurs in parks and protected areas, open water bodies and riparian buffer zones, which we excluded from the simulations.

The DEMAND submodel estimates per capita land consumption for a given area (e.g. county) based on the relationship between historical population totals and amounts of developed land across multiple reference dates (2001, 2004, 2006, 2008, 2011, 2013, 2016, 2019; see “Data Sources” below). The historical estimates are used to create a logarithmic trend function specific to, for example, each county in the study region. Future annual per capita land consumption is extrapolated for each county using annual population projections through the year 2050 based on a moderate socio-economic scenario (Shared Socioeconomic Pathway 2; SI Appendix, Table S2).

The PGA submodel allocates simulated growth using an iterative, stochastic, site selection process designed to mimic the urban forms (i.e., the spatial configuration of development patterns) characteristic to the study area. We parameterized the PGA submodel based on observed patterns of new development during the 2001–2019 reference period. We assumed the persistence of factors that drove the spatial configuration of development during the reference period throughout the projection phase. Using the GRASS GIS r.future.calib^70^ module, we generated a “patch library” with a distribution of patch sizes and shapes that are stochastically allocated during simulations.

### FUTURES 3.0 validation

We validated hindcasts of land change following accuracy assessment methods recommended by^71,72^. Specifically, we calculated allocation and quantity errors and assessed accuracy using the "figure of merit," a metric that measures the statistical agreement between observed and simulated changes within a defined area (SI Appendix, Figs. S5 and S6). Model performance is comparable to previous implementations of the FUTURES framework^29,56,60^ and other land change models^66,71^. However, quantitative evaluation of adaptive response outcomes was infeasible due to data scarcity. To our knowledge, no local efforts here or elsewhere in the U.S. have been adopted to systematically document ongoing adaptation measures or residents’ adaptation preferences based on metrics of adaptive capacity. Furthermore, FEMA’s national dataset on hazard-mitigated properties^73^ recorded only 25 properties in the study area that had taken action between 1999 and 2019 to reduce or eliminate long-term risk of flooding (e.g., floodproofing, elevating property, acquisition/demolition), a gross underestimate for an area at the leading edge of climate adaptation response. Residents and communities may have received support from alternative mitigation and recovery assistance programs (e.g., U.S. Department of Housing and Urban Development’s Community Development Block Grant, U.S. Small Business Administration’s Disaster Loan Assistance), but these data were unavailable for our analysis. To gain insight into the validity of the FUTURES 3.0 flood response function, we evaluated the correlation between social vulnerability^68,69^ (the CDC Social Vulnerability Index [SVI]) and FEMA’s redacted insurance claims^74^ processed from 2000–2009 and 2010–2022 (see SI Appendix, Figs. S7 and S8). We found significant, negative correlations (Pearson coefficient of − 0.24 [p < 0.05] for 2000–2009 and − 0.32 [p < 0.001] for 2010–2022) between the SVI score and the number of processed insurance claims for both time periods, which supports our assumption that less vulnerable census tracts are more likely to rebuild and enact in-situ adaptation measures.

### Adaptive response

We developed a plausible flood response function that relates adaptive capacity and level of flood damage sustained. This function delineates response state spaces (stay trapped, retreat, or protect and armor; Fig. 5B) based on published responses of humans to flood events^11,27,39–45^. To our knowledge, no frameworks or datasets are available that describe the actual choices or preferences of residents of the three-county test case location (South Carolina) in response to flood hazard and damage. We used the CDC Social Vulnerability Index (SVI^68,69^) to determine vulnerability at the census tract level, using the range of vulnerability as a metric of adaptive capacity (x-axis in Fig. 5B). The SVI takes into account parameters including socioeconomic status (e.g., poverty and unemployment rates, education levels), household characteristics (e.g., age distribution, disability rates, single-parent households), racial and ethnic minority status, and housing type and transportation factors (household structural attributes, crowding, vehicle availability)^69^. To implement the SVI scores^69^ (0 [lowest vulnerability] to 1 [highest vulnerability]) as metrics of adaptive capacity, we transformed the scores to range from − 1 (highest vulnerability) to 1 (lowest vulnerability). Each 30-m pixel is assigned the transformed SVI value of the census tract to which it belongs. We used depth-damage functions^75^ and the HAZUS General Building Stock^76^ to relate flood depth to percentage damage to structure value at the census block-level (y-axis in Fig. 5B). Depth-damage functions vary spatially depending on the distribution of building types (e.g., single floor dwelling, condominium, mobile homes, school, retail) in each census block. Regardless of adaptive capacity value, we assume that retreat is necessary above a user-defined threshold of damage (y-axis in Fig. 5B); damage that exceeds 50% of the structure’s value is known to require demolition and rebuilding^77^. For the “reactive” response function we set a 50% damage threshold value for an adaptive capacity value of 0, with higher thresholds for less vulnerable census tracts (75% of structure value) and lower for more vulnerable tracts (25%).

The flood response function determines whether the residents of a pixel leave the pixel (response is retreat) or remain (response is either stay trapped or protect and armor). The response for all pixels with values above the response function is “retreat” (the model does not distinguish between forced or voluntary retreat; Fig. 5B). Pixels with values below the response function but with positive adaptive capacity values are assumed to “protect and armor,” while those with negative adaptive capacity values (i.e., more vulnerable) are assumed to “stay trapped.”

However, because FUTURES is probabilistic, each pixel faces a stochastic challenge using two Gaussian distribution functions to ultimately characterize its likely flood response. For example, a pixel with a damage value of 0.51 and adaptive capacity value of 0.60 is highly likely to “protect and armor” because it falls below the flood response function (Fig. 5B), but there is also a low-probability chance of retreat given the Gaussian distribution.

At each time step of the simulation, the response of a developed pixel (stay trapped, retreat, or protect and armor) is determined by whether that pixel is simulated to flood, the percent damage incurred following a simulated flood event, and its adaptive capacity. Using a Monte Carlo approach, we stochastically simulate pseudo-flood events based on spatial flood probability data^38^. To characterize the spatial distribution of a flood event and account for hydrological connectivity between pixels, we stochastically simulate flood events within the boundaries of 12-digit Hydrologic Unit Codes (HUC-12)^78^. When a flood event in a particular HUC-12 occurs, the response of each flooded pixel is determined by the shape of the flood response function.

When a pixel’s outcome is “protect and armor,” we assume that a local-level (i.e., “pixel-level”) investment is made to enact protection (e.g., elevate homes or build a levee), which remains in place for all future time steps. If in a subsequent time step, the pixel experiences a less severe flood event, the pixel will remain unaffected. However, if that pixel experiences a more severe flood event, the past protect and armor response will be considered inadequate, and the flood response function and stochastic challenge will be used to determine the pixel’s response for that time step.

When a pixel’s outcome is “retreat,” we simulate new development elsewhere to accommodate population displacement (either in a different area of the same county, a different county within the study area, or a county outside of the study area); new development adds to the population quota in the DEMAND submodel of FUTURES for the subsequent time step. When a pixel response is “retreat,” development in that pixel is not allowed in future time steps and the pixel is marked as “abandoned.”

To simulate retreat, we used between-county migration values based on the Internal Revenue Service’s (IRS’s) annual series of county-to-county migration flow data for the years 1990–2015^79^. This dataset is the largest, most comprehensive record of county-to-county migration in the U.S. The IRS does, however, suppress migration flows comprising fewer than 10 individual migrants, systemically suppressing small rural migration flows.

Since the IRS dataset does not contain within-county migration information, we estimated the diagonal of the migration matrix to represent the probability that people relocate within the same county. We computed the net migration rate (normalized to range − 1 to 1) as a proxy for the “attractiveness” of a county. If net migration is zero, we set the within-county migration probability to 50%, meaning it is equally probable that a displaced pixel will be relocated within the same county versus to a different county. A net migration value of 1 then corresponds to a 100% chance of relocation within the county and a value of − 1 corresponds to a 100% chance of relocation to a different county. SI Appendix, Table S4, provides a subset of all origin/destination probabilities for study counties in South Carolina. Space limitations prevent displaying the full migration matrix (3 by 3152; where 3152 represents the total number of counties in the U.S.) for the study area (see Data and Code Availability for complete dataset). SI Appendix, Table S1, provides the likely destinations of residents from the three-county study area that resettled within South Carolina or another state due to retreat.

When relocating a pixel, we randomly select a county within the U.S., using the migration probabilities as weights. To adjust for the different population densities between origin and destination counties within the study area, we multiply the size of the pixel area (30 × 30 m, or 900 m^2^) by a ratio of the population density of the pixels in the origin county (numerator, known from DEMAND submodel) and the population density of the pixels in the destination county (denominator, known from the DEMAND submodel). In this way, if the origin pixel is from a more densely populated county than pixels in the destination county, the population from that one pixel is spread among multiple pixels in the destination county. The adjusted number of pixels is then added to the DEMAND table for the destination county for the next time step.

“Stay trapped” is the least likely response in our simulations, but we included this possible outcome in FUTURES 3.0 to make the model as generalizable as possible and to represent the reality of individuals being trapped by flooding^80^. Given the high social and economic costs of migration and adaptation^53^, “trapped” individuals are characterized by a lack of resources, skills, and/or desire to leave “at-risk” locations and a concurrent inability to protect their properties from flood damage, likely leading to a cycle of declining property values and quality of life^27^; these individuals may not migrate until assistance is provided or faced with a life-threatening situation. The difference between “stay trapped” and “protect and armor” in the model is dependent on adaptive capacity, assuming that the trapped pixels enact no adaptive measures and remain vulnerable to subsequent flood events.

### Development of the modeling approaches

All three modeling approaches described above (“Modeling Approach” section) share a baseline set of data: flood hazard conditions in 2020 and land cover in 2019. They also share the same flood hazard projections for 2035 and 2050. They differ in how they project the development footprint––either no new development (static development), urbanization that does not respond to flooding (dynamic development), or urbanization that does respond to flooding using the flood response function (climate-aware development). The latter two approaches share the same parameterization for the DEMAND, POTENTIAL, and PGA submodels. Only the “climate-aware development” approach uses the CLIMATE FORCING submodel. All three modeling approaches output the estimated amount and location of development by 2035 and 2050 (which is simply unchanged from 2019 for the “static development” approach). A negative change in developed land is possible for only the “climate-aware” modeling approach (due to “retreat” responses of pixels).

### Development of “what-if” policy interventions and flood response scenarios

The flexibility of FUTURES 3.0 (Fig. 5A) and the flood response function (Fig. 5B) accommodates creating different scenarios and altering the shape of the function to represent the inclinations of different communities or the influence of policies (Fig. 4A). We evaluated four additional response functions besides “reactive” [Fig. 4A(i)] to demonstrate how each influences estimates of exposed development. In the “managed retreat” scenario, policymakers incentivize converting developed land to undeveloped land (i.e., abandoned), with residents of all levels of adaptive capacity moving elsewhere [Fig. 4A(ii)]; the response function is altered to reduce the probability of both “stay trapped” and “protect and armor.” In the “resist” scenario, residents of all levels of adaptive capacity are highly resistant to retreat [Fig. 4A(iii)]; the response function is therefore altered to reduce the probability of “retreat.” In a “polarized population” scenario, both the least and most vulnerable residents remain in place, while those of intermediate adaptive capacity retreat [Fig. 4A(iv)]; the response function is altered to reflect a high probability of “protect and armor” for the least vulnerable residents and a high probability of “stay trapped” for the most vulnerable ones. Finally, in the “trapped population” scenario, vulnerable communities with little to no ability to adapt remain trapped in at-risk locations [Fig. 4A(v)], and the response function is altered to reduce the “retreat” outcome for vulnerable communities.

### Data sources for exposure analysis

#### Flood hazard

To establish current and future flood hazard conditions, we used nationwide data from First Street Foundation (FSF^38^) that differentiate flood depth based on the different return periods of flood events (the chance that a flood would occur in any given year): 2-year return period (50% annual chance of flooding), 5-year (20%), 20-year (5%), 100-year (1%), and 500-year (0.2%). FSF provides property-level flood hazard and water depth estimates as points at parcel centroids. To obtain estimates for undeveloped areas, where point data were not available, we primarily used FSF tiled data.

To disaggregate and refine FSF water depth levels > 1.2 m (values which were aggregated in a single category in the tiled data), we obtained the elevation of individual properties from a 1 arc-second U.S. Geological Survey (USGS) Digital Elevation Model (DEM^81^) and the water depth from FSF parcel-level flood data; we combined these two data sources to determine water surface elevation above sea level during a flood event. We then interpolated parcel-level water surface elevation into a 30-m raster and subtracted the DEM to obtain more precise water depth values across the terrain. We repeated this process for the FSF 2020, 2035, and 2050 datasets and each of the return periods (5-, 20-, 100-, 500-year flood) at each time interval. All of these datasets represent the “middle-of-the-road” climate change Representative Concentration Pathway (RCP) 4.5.

We used FSF data because FSF has developed one of the most sophisticated models that incorporate the effect of climate change on the spatial and temporal distribution of combined fluvial, pluvial, and coastal flood hazard. The evolution of the modeling methods has been validated by several prominent experts^38,82,83^. However, FUTURES 3.0 is flexible and can accommodate any kind of flood hazard data, such as the publicly available U.S. Federal Emergency Management Agency’s 100-year floodplain.

#### Land cover

We used USGS National Land Cover Database (NLCD^84,85^) data from 2001, 2004, 2006, 2008, 2011, 2013, 2016, and 2019 to parameterize the POTENTIAL, DEMAND, and Patch-growing Algorithm (PGA) submodels within FUTURES (see^29,59,65^ for details about parameterizing and calibrating FUTURES submodels; see SI Appendix, Tables S2 and S3, for details about predictor variables and the model coefficients used in this study). We used NLCD 2019 data to identify the baseline locations of developed land at the start of our simulations, overlaying flood hazard to understand development exposed to flooding.

We used NLCD data because they are publicly available with nationwide coverage, and widely used and vetted for a range of research applications. However, any land cover data can be used with FUTURES 3.0, including other established datasets or those newly classified from remotely sensed imagery.

### Supplementary Information

Supplementary Information.

## Data Availability

The data that support the findings of this study are available in Zenodo with the identifier https://doi.org/10.5281/zenodo.6607860.

## Code availability

The open source FUTURES software, including all submodels (POTENTIAL, DEMAND, PGA, CLIMATE FORCING), can be accessed through the GitHub repository (https://github.com/ncsu-landscape-dynamics/GRASS_FUTURES) with the identifier https://doi.org/10.5281/zenodo.6607097.
