# Supplementary material for: Spatially interactive modeling of land change identifies location-specific adaptations most likely to lower future flood risk
Source: Sci Rep. 2023 Nov 1;13:18869. doi: 10.1038/s41598-023-46195-9 (PMC10620417; doi:10.1038/s41598-023-46195-9)
Supplement: Supplementary file 1 — Supplementary Information. [file 41598_2023_46195_MOESM1_ESM.pdf]

Spatially interactive modeling of land change identifies location-specific adaptations most likely to lower future flood risk

Georgina M. Sanchez[1,*], Anna Petrasova[1], Megan M. Skrip[1], Elyssa L. Collins[1], Margaret A. Lawrimore[1], John B. Vogler[1], Adam Terando[2,3], Jelena Vukomanovic[1,4], Helena Mitasova[1,5], Ross K. Meentemeyer[1,6]

[1]Center for Geospatial Analytics, North Carolina State University, Raleigh, NC, United States of America
[2]U.S. Geological Survey, Southeast Climate Adaptation Science Center, Raleigh, NC, United States of America
[3]Department of Applied Ecology, North Carolina State University, Raleigh, NC, United States of America
[4]Parks, Recreation and Tourism Management, North Carolina State University, Raleigh, NC, United States of America
[5]Department of Marine, Earth and Atmospheric Sciences, North Carolina State University, Raleigh, NC, United States of America
[6]Department of Forestry and Environmental Resources, North Carolina State University, Raleigh, NC, United States of America

[*]Corresponding author: Georgina M. Sanchez.
Email: gmsanche@ncsu.edu

Table of Contents:

Supplementary Text
Figures S1 to S8
Table S1 to S4
References

Supplementary Text

Modeling Approaches. We estimated exposure of urban development to future flood hazard using three modeling approaches: static development, dynamic development, and climate-aware development. Figure S1 provides a visual schematic and description of each approach.

Additional Results. Table S1 provides the likely destinations of displaced pixels from the three-county study area that resettled within South Carolina or another state due to a simulated outcome of “retreat” using a climate-aware modeling approach for a “reactive” response function.

Figure S2, S3, and S4 provide results associated with the business-as-usual “reactive” flood response function and the four additional response functions (i.e., managed retreat, resist, polarized population, trapped population) computed using the climate-aware modeling approach. Figure S2 shows projections of simulated outcomes of new development, retreat, protect and armor, and stay trapped by response scenarios and simulation period (i.e., 2033 and 2050). Figure S3 shows the estimated cumulative developed land area within different hazard zones (50%, 20%, 5%, 1%, and 0.2% annual chance of flooding) over time. Figure S4 shows percentage change in developed land exposed to future flooding (annual flood probability of 0.2% by 2050) by census tract.

FUTURES Parameterization. Geospatial predictor variables, their descriptions, and the data sources used to parameterize FUTURES submodels during the reference period (2001–2019) are detailed in Table S2. Mixed-effects model coefficient estimates included in the POTENTIAL submodel are detailed in Table S3. We computed 50 stochastic iterations at annual timesteps for the simulation period 2020–2050. Step-by-step instructions to parameterize and compute simulations with FUTURES in GRASS GIS are available at: https://grass.osgeo.org/grass-stable/manuals/addons/r.futures.html.

FUTURES Validation. To evaluate model accuracy of simulated patterns of land change, we followed a hindcast approach—predicting or recreating past conditions of land change. We parameterized the FUTURES 3.0 model using observed new development identified during the validation reference period (2001–2008) and assessed model accuracy for the validation simulation period (2009-2019). Simulations for 20 stochastic iterations used the climate-aware development modeling approach under a “reactive” response scenario and assumed baseline (2020) flood hazard conditions.

Arguably, it is as important to accurately simulate land changes as it is to simulate persistence (i.e., null successes or locations of no change between 2009–2019). However, accuracy assessments primarily focused on simulated persistence result in overconfidence in model performance due to the predominantly static nature of most landscapes[1,2]. Therefore, we based model performance on the accuracy of simulated patterns of land changes at the end of the simulation time period (i.e., 2019), and we quantified accuracy using metrics of quantity error and allocation error[3,4]. Quantity error measures disagreements between the total amount of simulated and observed change, whereas allocation error evaluates whether simulated changes happened in the same locations as observed changes. Pixels that represent the simulated outcome of “retreat” are assumed to maintain impervious surface characteristics, therefore, we treated these locations as developed land for validation purposes. Thus, for land change validation, we make no distinction between abandoned or inhabited developed land.

To evaluate FUTURES’ ability to simulate patterns of new development across the urban-rural gradient of the three-county study area, we first imposed 6 x 6 km grid cells over the area and estimated development density for each cell as of 2008. We then calculated the simulation accuracy for each 6 x 6 km cell in 2019 using the “figure of merit” (FoM[3]; see equation 1) metric as follows:

$$\text{Figure of Merit} = B / (A + B + C + D) \qquad (1)$$

The FoM[3] quantifies the statistical agreement between observed and simulated changes for a given area, differentiating between incorrectly simulated persistence (misses; A), correctly simulated change (hits; B),

and incorrectly simulated change (false alarms; *D*). FoM values can range 0–100%, where 0% represents no match or overlap between simulated and observed change, and 100% represents a perfect match. The FoM also differentiates error associated with an incorrectly predicted category (*C*), however, this applies to models that, unlike FUTURES, simulate changes in more than one land-use/land-cover category.

Simulations projected that 2.06% of the study area changed, while observed change was 1.06% of the region (Fig. S5A). We correctly simulated 88.74% of landscape outcomes, however, 88.67% was correctly simulated persistence (i.e., null successes or no change) and 0.07% was correctly simulated change (i.e., hits; Fig. S5A). Additionally, we partitioned initially developed land (i.e., 8.28% of the landscape by 2008) to reflect model assumptions that prevent FUTURES from simulating new growth in already developed areas. Because the proportion of false alarms (1.99%) is greater than the proportion of misses (0.99%), the simulations are considered to overestimate growth across the study region. We further examined total error by type and found that 1.99% is allocation error and 0.99% is due to quantity disagreements (Fig. S5B).

FoM estimates, calculated for each 6 x 6 km grid cell and averaged for the 20 stochastic simulations ranged from 0–35% across the study area (Fig. S6). We found better agreement between simulated and observed patterns of land change across more densely developed urban and suburban areas than in the rural areas (Fig. S6). Similar to previous studies[1,3], we found that less densely developed rural areas have lower estimates of FoM, as it is more challenging for models to correctly simulate small net changes that are more dispersed and isolated.

Validation of adaptive response outcomes (i.e., stay trapped, retreat, or protect and armor) was deemed infeasible due to scarcity of observed data for comparison. To our knowledge, no available dataset provides sufficient historical records of response choices to flood hazards and damages across the study area.

Relationship Between Social Vulnerability and Insurance Claims. To draw insights into how social vulnerability relates to adaptive flood response, we assessed the relationship between the Centers for Disease Control and Prevention's Social Vulnerability Index (SVI[5]) and the Federal Emergency Management Agency's (FEMA) redacted insurance claim transactions[6]. Recent estimates of social vulnerability across the three-county study area show a 21% decrease in vulnerability scores from 2000 to 2010 (Fig. S7A–B). The CDC's SVI scores range from 0 (lowest vulnerability) to 1 (highest vulnerability); the average SVI score was 0.53 in 2000 and 0.42 in 2010. Recent records of insurance claims show that residents across the three-county study area processed 713 claims from 2000 to 2009 (Fig. S7C; average covered cost to building damage of $14,065) and 6,747 claims from 2010 to 2022 (Fig. S7D; average covered cost of $23,009). Overall, we found significant, negative correlations (Pearson r coefficient of -0.24 [$p < 0.05$] for the 2000–2009 period, and -0.32 [$p < 0.001$] for the 2010–2022 period) between vulnerability and the number of processed insurance claims for both time periods (Fig. S78–B). These results suggest that less socially vulnerable residents (i.e., low SVI scores) are more likely to rebuild and repair damage from floods and pursue flood mitigation efforts such as elevation certificates.

Migration Matrix. Table S4 provides estimated county-to-county migration probabilities for the three-county study counties in South Carolina. This represents a subset from the full 3 by 3,152 migration matrix which includes the total number of counties in the U.S. (i.e., 3,152). The complete dataset is available in Zenodo with the identifier doi:10.5281/zenodo.6607860 (see Data and Code Availability).

Supplementary Figures

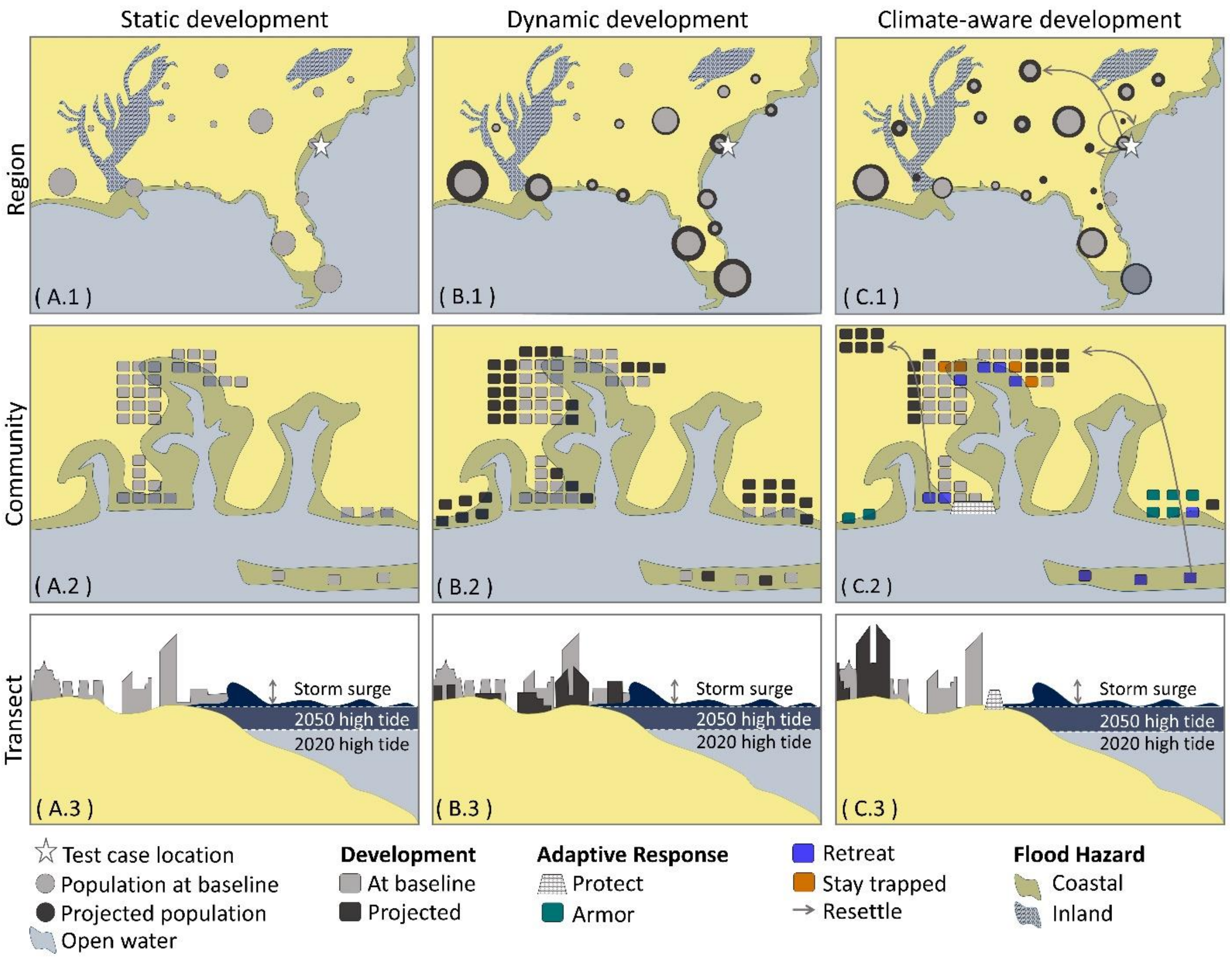

Fig. S1. Modeling approaches to project the exposure of urban development to future flood hazard (Columns A–C) illustrated at different spatial scales and views: regional (row 1), coastal top-down view (row 2), and local transect (row 3). The *static development* approach assumes no population growth or urbanization into the future (A.1, A.2, A.3); exposure is based on future flood hazard and does not account for future growth. The *dynamic development* approach accounts for future population growth and urbanization, but no adaptive response to flooding is considered while estimating exposure (B.1, B.2, B.3). The *climate-aware development* approach integrates all three components of flood risk: exposure (patterns in urban development within floodplains), hazard (increases in flooding due to climate change), and vulnerability (capacity for adaptive response) (C.1, C.2, C.3).

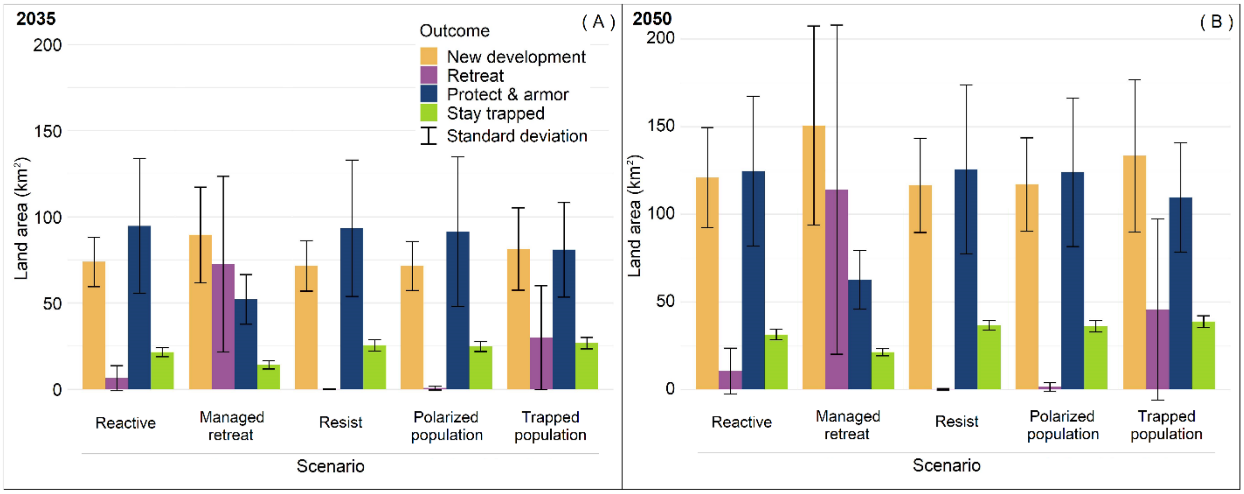

Figure S2. Projections of simulated outcomes of new development, retreat, protect and armor, and stay trapped for five adaptive response scenarios and two simulation years (2035, 2050) across the three-county test case region in South Carolina. Average land area values and standard deviations derived from 50 stochastic model iterations computed using the climate-aware modeling approach.

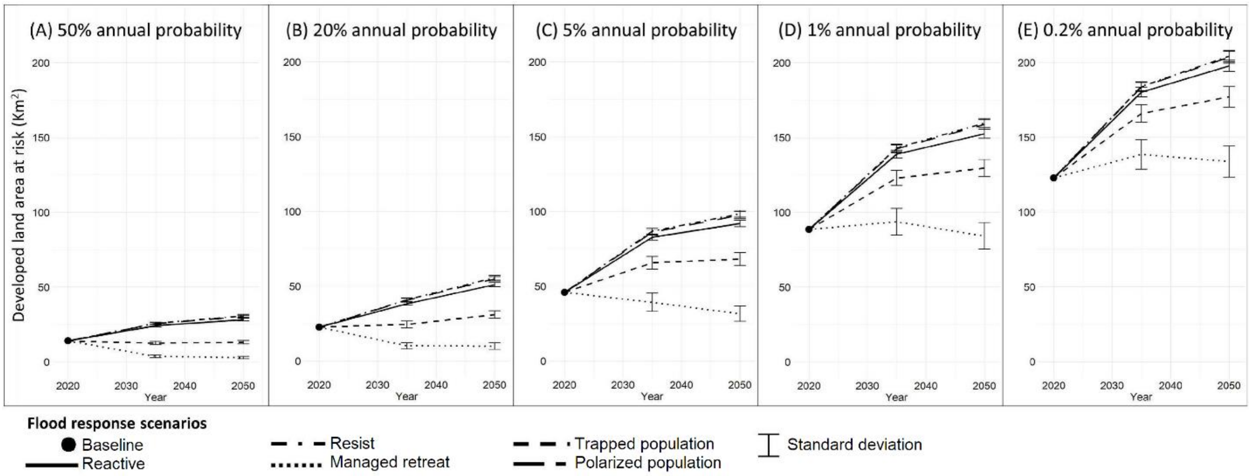

Fig. S3. Estimated cumulative developed land area within different hazard zones computed using the FUTURES 3.0 climate-aware development modeling approach. The hazard zones are 50% (2-yr floodplain; A), 20% (5-yr floodplain; B), 5% (20-yr floodplain; C), 1% (100-yr floodplain; D), and 0.2% (500-yr floodplain; E) annual chance of flooding. We modeled five adaptive response scenarios, i.e., reactive , managed retreat, resist, polarized population, and trapped population. Baseline conditions (i.e., 2019 development, 2020 flood hazard) are displayed as a filled circle in the graphs for reference. Standard deviations were derived from 50 stochastic urban growth simulations.

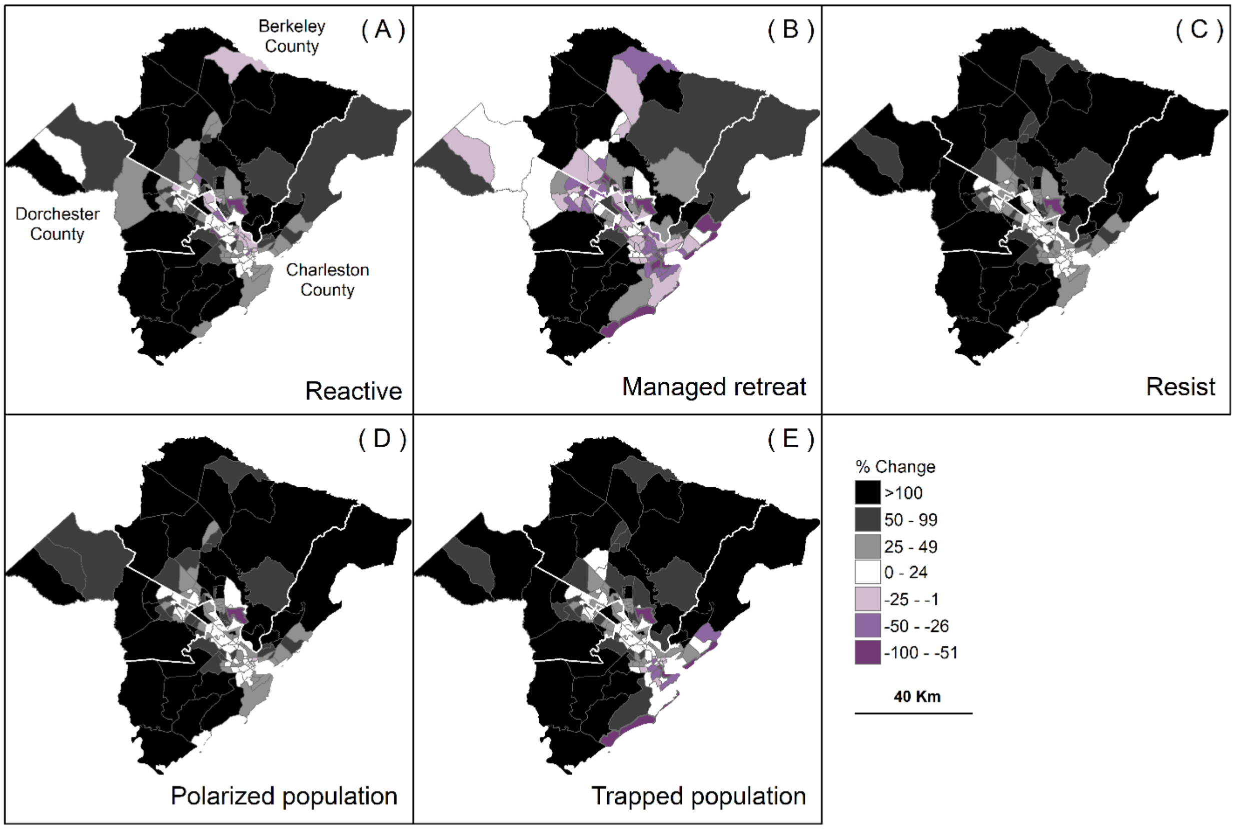

Fig. S4. Percentage change in developed land exposed to future flooding (i.e., annual flood probability of 0.2% [500-yr floodplain] by 2050) by census tract. All projections computed using the climate-aware development modeling approach. We modeled five adaptive response scenarios, i.e., reactive (A), managed retreat (B), resist (C), polarized population (D), and trapped population (E). Percentage change was calculated as relative to baseline conditions (i.e., 2019 development, 2020 flood hazard).

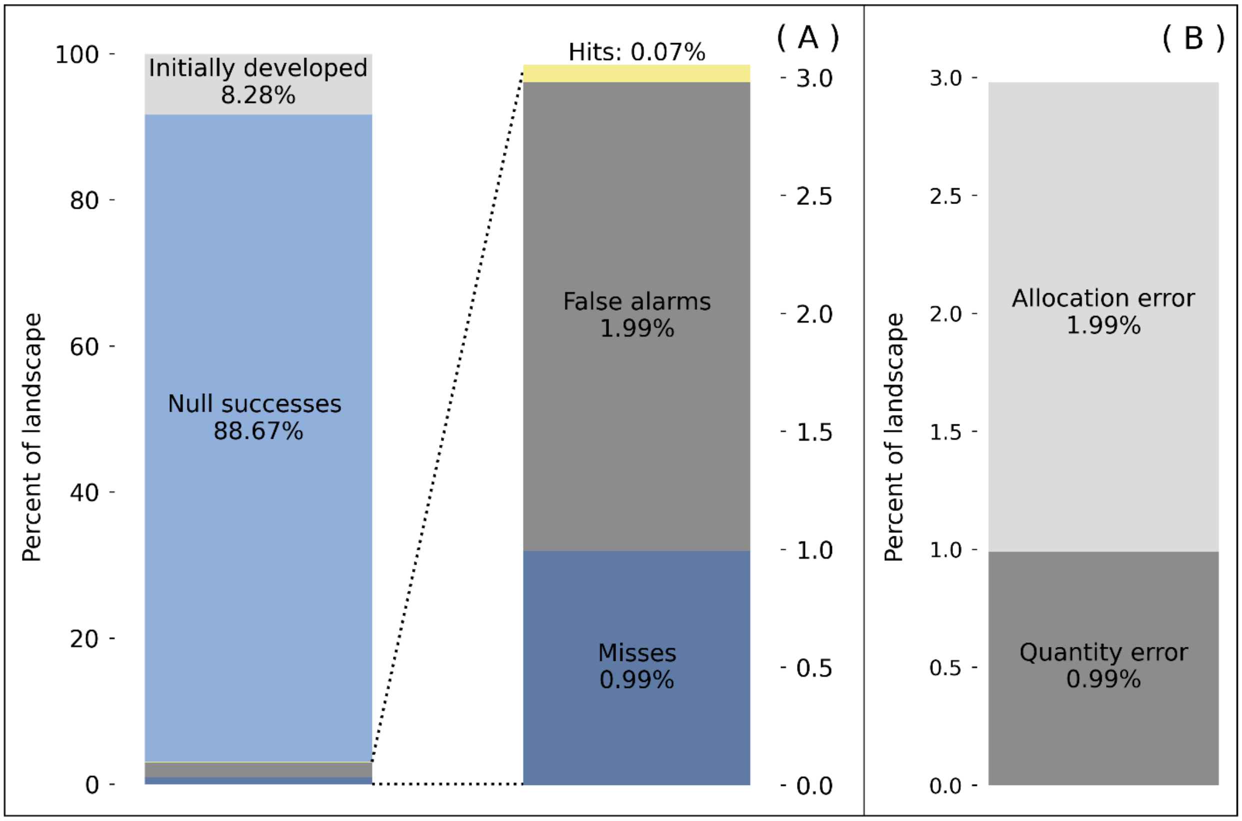

Fig. S5. Proportion of simulation successes and errors for the study landscape (A) partitioned by error type (B). Initially developed land (i.e., by 2008) is partitioned to reflect model assumptions that prevent FUTURES from simulating new growth in developed areas. Null successes refer to observed persistence simulated as persistence. Hits refer to observed change simulated as change. False alarms refer to observed persistence simulated as change. Misses refer to observed change simulated as persistence. Quantity error quantifies the disagreement between the total amounts of simulated and observed change. Allocation error is the percentage of landscape where the locations of simulated and observed changes differ. Values derived from 20 stochastic urban growth simulations computed using the climate-aware modeling approach in a “reactive” response scenario.

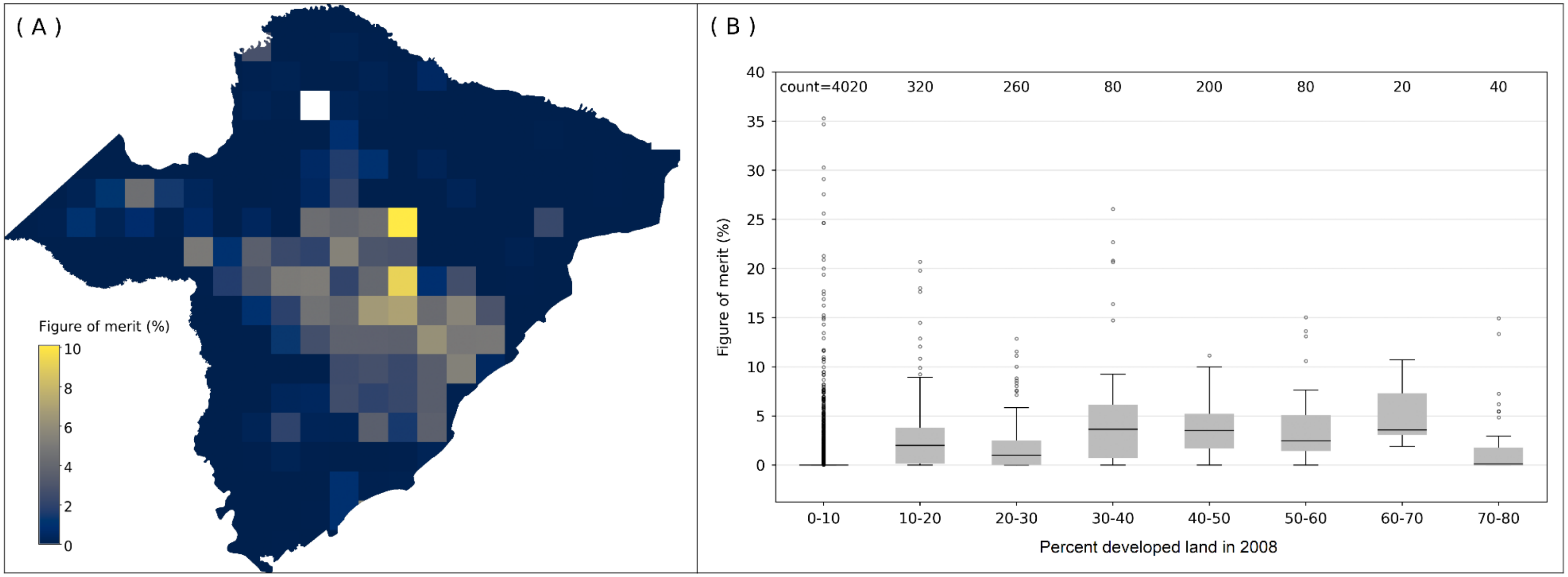

Fig. S6. Simulation accuracy estimated using the figure of merit (FoM) calculated and averaged for each 6 x 6 km grid cell in the study region (A) and plotted by development density (B). FoM assesses model performance by quantifying the statistical agreement between observed and simulated change. FoM values averaged from 20 stochastic urban growth simulations computed using the climate-aware modeling approach in a "reactive" response scenario. The box-plot (B) displays lower (25th percentile) and upper (75th) quartile boundaries, median (line inside the box), lower (10th) and upper (90th) error lines, and values outside 10th and 90th percentiles (circles).

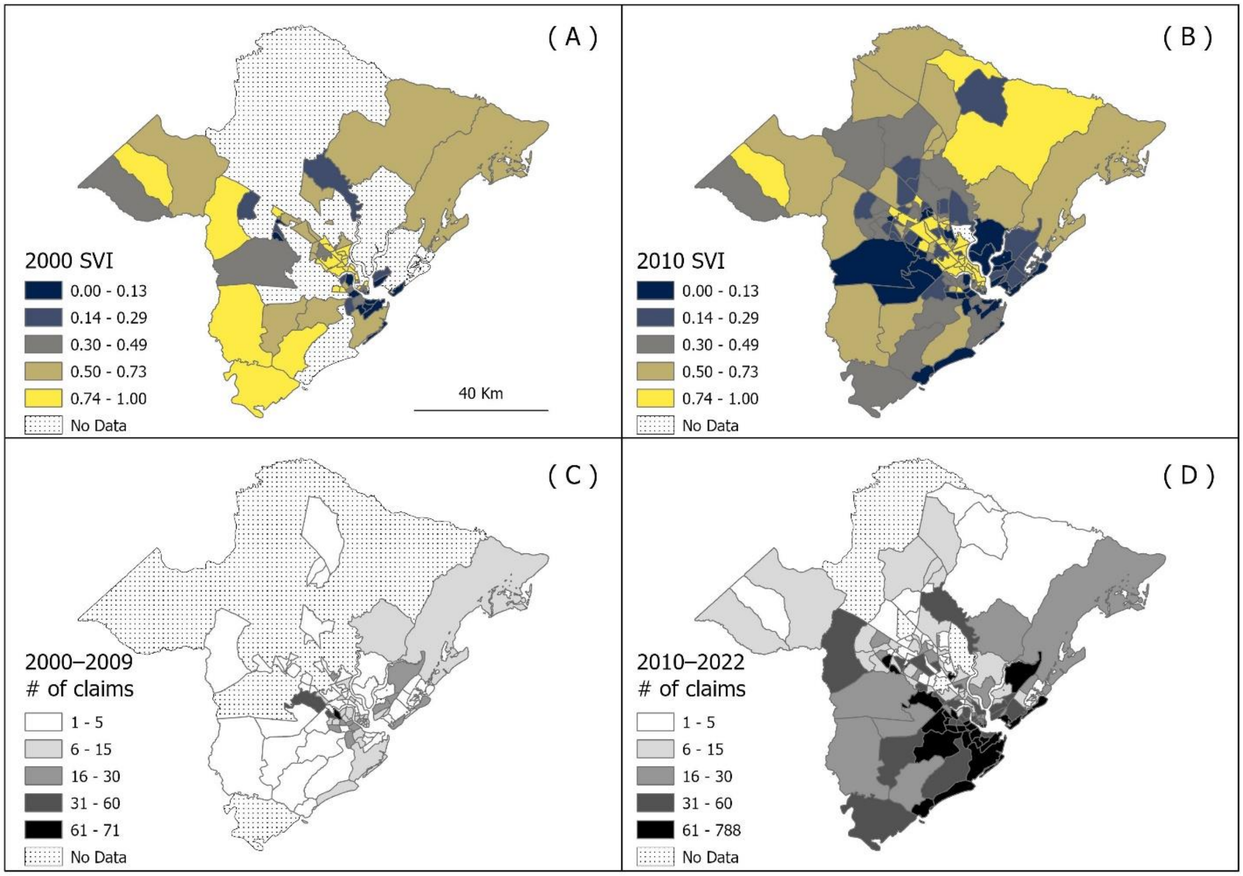

Fig. S7. Estimated Social Vulnerability Index (SVI) in 2000 (A) and 2010 (B) and the number of redacted insurance claim transactions between 2000–2009 (C) and 2010–2022 (D). Values shown for census tracts. SVI values range from lowest (0) to highest (1) vulnerability.

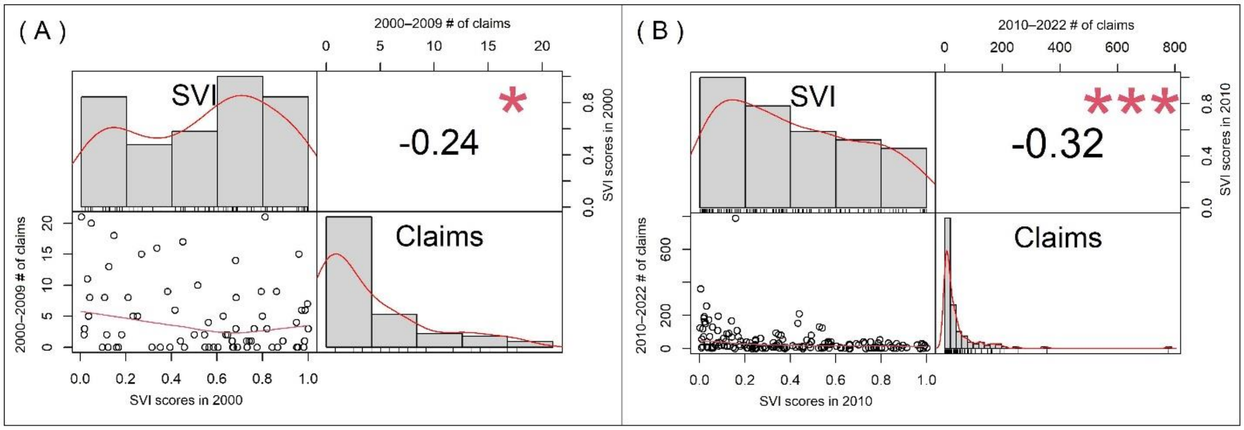

Fig. S8. Correlation matrix of the Social Vulnerability Index (SVI) and the number of redacted insurance claim transactions between 2000–2009 (A) and 2010–2022 (B). Estimates of SVI represent socioeconomic conditions in 2000 (A) and 2010 (B) census tracts. Diagonals display distributions of values, the lower left corners display bivariate scatter plots with fitted line, and the upper right corners show the Pearson correlation coefficient and significance level (*** p<0.001; * p<0.05).

Supplementary Tables

Table S1. Likely destinations of residents from the three-county study area that resettled within South Carolina (SC) or another state due to “retreat.” Numbers of displaced pixels are averaged across the 50 stochastic urban growth simulations computed using the climate-aware modeling approach in a “reactive” response scenario. States are ordered top to bottom by increasing distance from the study area. An average of 11791±1860 total pixels experienced retreat. The average number of total abandoned pixels that relocated outside the study area was 2842.6±470.4.

Destination	Displaced pixels	Variability (±)
Within study area	8949.4	1389.3
*South Carolina	764.6	120.5
Georgia	280.68	47.5
North Carolina	292.82	51.6
Virginia	257	46.5
Florida	334.64	61.6
West Virginia	0.68	1
Alabama	27.24	8.1
Tennessee	59	13.7
Kentucky	16.68	5.6
District of Columbia	17.66	5.6
Maryland	58.46	13.7
Delaware	4.92	2.2
Mississippi	10.98	3.4
Ohio	38.68	10.1
New Jersey	18.08	4.9
Pennsylvania	32.64	7.8
Indiana	5.5	2.7
Arkansas	2.4	1.6
Connecticut	49.32	10.9
Louisiana	13.58	4.6
Illinois	36.32	8.5
Missouri	6.52	2.2
Rhode Island	7.22	2.7
New York	100.82	18.4
Massachusetts	21.3	6.7
New Hampshire	1.54	1.2
Michigan	12.28	3.5
Wisconsin	1.08	1.1
Vermont	0.2	0.4
Oklahoma	9.4	3.8
Maine	1.02	0.9
Kansas	1.1	0.9

Texas	92.98	18.2
Minnesota	2.3	1.7
Nebraska	2.74	1.9
New Mexico	2.82	1.9
Colorado	22.24	6.9
Utah	1.98	1.3
Arizona	26.42	6.8
Nevada	13.54	4.5
California	109.68	22.8
Oregon	4.38	2
Washington	79.16	17.4

*Average number of displaced pixels that stayed within SC and outside of the study area.

Table S2. Future Urban-Regional Environment Simulation (FUTURES) submodel predictor variables, descriptions, and sources.

Submodel	Predictor	Description	Base data	Year(s)	Data source
**POTENTIAL**					
	Land cover	Land classified as developed, forest, planted/cultivated, wetland	NLCD	2001, 2011, 2016, 2019	[7,8]
	Roads	Euclidean distance to roads	TIGER/Line Shapefiles	2019	[9]
	Topography	Elevation and slope	National Elevation Dataset	2011	[10]
	Hydrography	Euclidean distance to surface water bodies and coast	Lakes, rivers, reservoirs, coast lines	2011	[11]
	Crop production	Average crop production	Crop Productivity Index derived from SSURGO	2021	[12]
	Multilevel structure	County boundaries	TIGER/Line Shapefiles	2019	[9]
	Development pressure	Number of nearby developed pixels within search distance and weighted by distance	Historical and projected development patterns	2001-2050	NLCD[7,8] and FUTURES simulations
	Social Vulnerability Index	SVI uses census data to determine the social vulnerability of every census tract	SVI	2018	[5]
**DEMAND**					
	Population	Historical and projected population	SSP2 projections	2001-2050	[13,14]
	Development	Land classified as developed and undeveloped	NLCD	2001, 2004, 2006, 2008, 2011, 2013, 2016, 2019	[7,8]

NLCD = National Land Cover Database; TIGER = Topologically Integrated Geographic Encoding and Referencing; SSURGO = Soil Survey Geographic Database; SVI = Social Vulnerability Index; FUTURES = FUTure Urban-Regional Environment Simulation; SSP2 = Shared Socioeconomic Pathways (Middle of the Road).

Table S3. Description of generalized linear mixed-effects POTENTIAL submodel (site suitability). Random effects vary by county and are calculated for intercept and development pressure.

Fixed effects	Coefficient	Std. error
Intercept ***	-5.08	0.38
Social Vulnerability Index ***	-1.41	0.18
Distance to roads ***	-0.51	0.03
Distance to forest land ***	1.05	0.03
Slope ***	1.07	0.13
Average crop production *	0.17	0.04
Distance to wetlands ***	-3.13	0.21
**Random effects**	**Variance**	**Std. dev.**
County (intercept)	-3.42	0.26
Development pressure	0.18	0.01

*** $p < 0.001$; * $p < 0.1$

Table S4. Subset of migration destination probabilities for counties in South Carolina, U.S.

Origin / Destination	**Berkeley**	**Charleston**	**Dorchester**
Abbeville	0.000	0.000	0.000
Aiken	0.180	0.258	0.267
Allendale	0.000	0.000	0.000
Anderson	0.168	0.221	0.174
Bamberg	0.000	0.008	0.025
Barnwell	0.000	0.010	0.000
Beaufort	0.362	0.442	0.453
**Berkeley**	65.789	4.136	7.779
Calhoun	0.000	0.022	0.000
**Charleston**	7.804	66.808	8.661
Cherokee	0.000	0.000	0.000
Chester	0.000	0.000	0.000
Chesterfield	0.000	0.000	0.000
Clarendon	0.163	0.102	0.108
Colleton	0.638	0.462	1.416
Darlington	0.012	0.099	0.000
Dillon	0.000	0.000	0.000
**Dorchester**	4.271	2.925	67.672
Edgefield	0.000	0.000	0.000
Fairfield	0.000	0.008	0.000
Florence	0.288	0.282	0.336
Georgetown	0.191	0.279	0.011
Greenville	0.530	0.577	0.803
Greenwood	0.004	0.104	0.007
Hampton	0.002	0.048	0.000
Horry	0.476	0.433	0.528
Jasper	0.000	0.029	0.000

Kershaw	0.021	0.081	0.037
Lancaster	0.000	0.035	0.000
Laurens	0.000	0.044	0.000
Lee	0.000	0.000	0.000
Lexington	0.603	0.481	0.851
McCormick	0.000	0.000	0.000
Marion	0.000	0.010	0.000
Marlboro	0.000	0.000	0.000
Newberry	0.000	0.039	0.000
Oconee	0.006	0.104	0.003
Orangeburg	0.719	0.348	1.333
Pickens	0.131	0.213	0.183
Richland	0.805	0.776	1.155
Saluda	0.000	0.000	0.000
Spartanburg	0.260	0.311	0.333
Sumter	0.190	0.202	0.161
Union	0.000	0.000	0.000
Williamsburg	0.105	0.045	0.000
York	0.251	0.305	0.365

Values represent county-to-county migration probabilities from 1990–2015 based on the Internal Revenue Services migration flow data[15]. Between- and within-county migration rates were computed for each county in the U.S. (see Methods). For visualization purposes, we display only South Carolina counties here. Study area counties are bolded.

References

1. Pontius, R. G., Shusas, E. & McEachern, M. Detecting important categorical land changes while accounting for persistence. *Agric Ecosyst Environ*, 101(2–3):251–268, https://doi.org/10.1016/j.agee.2003.09.008 (2004).
2. van Vliet, J. et al. A review of current calibration and validation practices in land-change modeling. *Environ. Model. Softw.*, 82, pp. 174-182, 10.1016/j.envsoft.2016.04.017 (2016).
3. Pontius, R. G. et al. Comparing the input, output, and validation maps for several models of land change. *Annals of Regional Science*, 42:11–37 (2008).
4. Chen, H. & Pontius, R.G. Diagnostic tools to evaluate a spatial land change projection along a gradient of an explanatory variable. *Landscape Ecology*, 25:1319–31 (2010).
5. Centers for Disease Control and Prevention (CDC). Social Vulnerability Index. Agency for Toxic Substances and Disease Registry, Geospatial Research, Analysis, and Services Program, https://www.atsdr.cdc.gov/placeandhealth/svi/index (2018).
6. Federal Emergency Management Agency (FEMA). OpenFEMA Dataset: FIMA NFIP Redacted Claims - v1. Available at https://www.fema.gov/about/openfema/data-sets (2022).
7. Homer, C. G. et al. Conterminous United States land cover change patterns 2001–2016 from the 2016 National Land Cover Database. *ISPRS Journal of Photogrammetry and Remote Sensing*, 162, p. 184–199, https://doi.org/10.1016/j.isprsjprs.2020.02.019 (2020).
8. Dewitz, J. & U.S. Geological Survey. National Land Cover Database (NLCD) 2019 Products (ver. 2.0, June 2021). U.S. Geological Survey data release, https://doi.org/10.5066/P9KZCM54 (2021).
9. U.S. Census Bureau. TIGER/Line Files and Shapefiles. Available at https://www.census.gov/geographies/mapping-files.html (2019).
10. U.S. Geological Survey (USGS). 1 Arc-second Digital Elevation Models (DEMs). USGS National Map 3DEP Downloadable Data Collection. Available at https://www.usgs.gov/3d-elevation-program (2017).
11. U.S. Geological Survey (USGS). National Hydrography Dataset. Available at https://www.usgs.gov/national-hydrography/access-national-hydrography-products (2019).
12. U.S. Department of Agriculture (USDA) Natural Resources Conservation Service (NRCS). National Commodity Crop Productivity Index. Available at https://ncsu.maps.arcgis.com/home/item.html?id=9ce0371b69564139b6d13264d2d46a31 (2021).
13. Hauer, M. E. Population projections for U.S. counties by age, sex, and race controlled to shared socioeconomic pathway. *Scientific Data*, https://doi.org/10.1038/sdata.2019.5 (2019).
14. The National Vital Statistics System (NVSS). U.S. Census Populations with Bridged Race Categories. Available at https://seer.cancer.gov/popdata/download.html (2021).
15. Internal Revenue Services (IRS). SOI Tax Stats Migration Data Files. Available at https://www.irs.gov/statistics/soi-tax-stats-migration-data (2020).
